# Effects of Radiation-Induced Skin Injury on Hyaluronan Degradation and Its Underlying Mechanisms

**DOI:** 10.3390/molecules28217449

**Published:** 2023-11-06

**Authors:** Jiahui Dong, Boyuan Ren, Yunfei Tian, Guanqun Peng, Huiting Zhai, Zhiyun Meng, Ruolan Gu, Hui Gan, Zhuona Wu, Yunbo Sun, Guifang Dou, Shuchen Liu

**Affiliations:** 1 Department of Pharmaceutical Sciences, Beijing Institute of Radiation Medicine, Beijing 100850, China; 17857314877@163.com (J.D.); 13821315006@163.com (B.R.); mengzhiyun@vip.163.com (Z.M.); gurl311@126.com (R.G.); ganh2003@163.com (H.G.); wznphd@126.com (Z.W.); sunyunbo0919@126.com (Y.S.); 2 School of Pharmacy, Henan University, Kaifeng 475004, China; tyf96599@163.com (Y.T.); zhaihuit@163.com (H.Z.); 3 College of Life Science, Hebei University, Baoding 071002, China; guanqun_peng@163.com

**Keywords:** radiation-induced skin injury, hyaluronan, degradation, mechanisms, *MEK5*/*ERK5* pathway, inflammatory factors

## Abstract

Radiation-induced skin injury (RISI) is a frequent and severe complication with a complex pathogenesis that often occurs during radiation therapy, nuclear incidents, and nuclear war, for which there is no effective treatment. Hyaluronan (HA) plays an overwhelming role in the skin, and it has been shown that UVB irradiation induces increased HA expression. Nevertheless, to the best of our knowledge, there has been no study regarding the biological correlation between RISI and HA degradation and its underlying mechanisms. Therefore, in our study, we investigated low-molecular-weight HA content using an enzyme-linked immunosorbent assay and changes in the expression of HA-related metabolic enzymes using real-time quantitative polymerase chain reaction and a Western blotting assay. The oxidative stress level of the RISI model was assessed using sodium dismutase, malondialdehyde, and reactive oxygen species assays. We demonstrated that low-molecular-weight HA content was significantly upregulated in skin tissues during the late phase of irradiation exposure in the RISI model and that HA-related metabolic enzymes, oxidative stress levels, the *MEK5*/*ERK5* pathway, and inflammatory factors were consistent with changes in low-molecular-weight HA content. These findings prove that HA degradation is biologically relevant to RISI development and that the HA degradation mechanisms are related to HA-related metabolic enzymes, oxidative stress, and inflammatory factors. The *MEK5*/*ERK5* pathway represents a potential mechanism of HA degradation. In conclusion, we aimed to investigate changes in HA content and preliminarily investigate the HA degradation mechanism in a RISI model under γ-ray irradiation, to consider HA as a new target for RISI and provide ideas for novel drug development.

## 1. Introduction

With the rapid development of technology, ionizing radiation has become widely used as a new energy source. However, radiation injury can occur in various applications of ionizing radiation. Owing to the penetrating and high-energy properties of radiation, it can dissociate electrons from molecules in the body, resulting in varying degrees of body damage [1,2]. Skin is the largest organ in the body and acts as a physical barrier against infectious pathogens and external injuries. Radiation-induced skin injury (RISI) can occur if the body is exposed to acute or chronic radiation [3]. To date, substantial progress has been achieved in understanding the mechanisms and treatment of RISI, and numerous medications are available worldwide for the prevention and treatment of RISI [4]. However, some limitations still remain. Owing to the lack of high-quality, large-sample studies and uniform evaluation criteria, the conclusions of various studies often contradict each other and lack generalizability. Therefore, it is vital to comprehensively explore the mechanism of RISI and conduct further research and development of targeted drug studies.

RISI is a common type of radiation complication in situations such as industrial nuclear accidents, occupational exposure, nuclear medicine exposure, and military environments [5,6]. RISI caused by ionizing radiation (such as X-rays and γ-rays) is also known as radiation dermatitis, which can be acute or chronic [7]. The basic pathogenesis of radiation dermatitis includes reactive changes in the skin capillaries after radiation exposure followed by vasodilation, local congestion, and erythema formation. Subsequently, the microvascular membrane swells, leading to spasms, narrowing and clogging of the lumen, and ulceration, or worse, fibrosis of the skin [8]. RISI can be further classified into three types of common skin injury—skin radiation injury, irradiation dermatitis, and burns caused by irradiation oncology surgery—based on differences in irradiation sources, exposure doses, disease course, and treatment methods [9]. The degree of RISI depends on the radiation type (e.g., α-, β-, γ-, and X-rays, and neutron radiation) and the irradiation dose received [10]. Different radiation sources are associated with various injury mechanisms to the skin, but pathologic manifestations of skin injury are somewhat similar [11]. In constructing animal models of RISI, most researchers use electron beams and γ- and X-ray irradiation to establish RISI models, but the irradiation dose, irradiation mode, and injury degree evaluation in the modeling process have not been standardized at present [12,13]. In this study, we selected γ-rays as the source of irradiation to investigate their effects on skin cells and tissues. This choice was based on the characteristics of γ-rays, which include a short range, the ability to increase the dose to the target area while significantly reducing the dose to the surrounding normal tissues, high safety and efficiency, and minimal irradiation injury [14].

Human keratinocyte (HaCaT) cells, an immortalized human keratinocyte cell line with a morphology similar to that of epithelial cells, are located in the upper layers of the skin and are the primary target cells for radiation injury as they have been extensively used in skin biology and differentiation research [15]. Moreover, based on references to the use of the HaCaT cell line to study the mechanism of RISI injury at different timepoints (24, 48, 72 h) in vitro, we selected the HaCaT cell line and five timepoints as a cell model for RISI [16,17,18]. In recent years, radiological and nanomaterial research has been at the forefront of scientific research [19], and many novel materials have been used to treat radiological injuries and have already had milestones of achievement [20]. Among them, hyaluronan (HA), a natural hydrophilic polymer, has been widely used as a raw material to prepare new materials for resistance to radiation injury [21]. HA is an unsulfated, linear unbranched glycosaminoglycan naturally occurring in the body, and is abundant in epithelial cells with a molecular formula of (C_14_H_21_NO_11_)_n_ and a molecular weight range of 1000–10,000 kDa, comprising disaccharide units of D-glucuronic acid and N-acetyl-D-glucosamine linked via β-1,3-glycosidic bonds [22,23,24]. It is an important component of the extracellular matrix of the skin [25], with approximately 50% of the HA in the body being present in the skin tissue. In addition, HA is present in other parts of the body, including the vitreous humor of the eye, the umbilical cord, synovial fluid, cartilage, thoracic lymph, urine, serum, and amniotic fluid [26]. The properties of HA are directly dependent on its molecular weight, with current research showing that high-molecular-weight HA exhibits anti-inflammatory and immunosuppressive properties and regulates biological functions such as cell proliferation, cell migration [24], wound healing [27], and angiogenesis [28] in the body, whereas low-molecular-weight HA is a potent proinflammatory molecule and can alter cell behavior and signaling. In vertebrates, the degradation balance of HA is controlled by hyaluronidase (HYAL), HA receptor (*CD44*) [29], inflammatory factors, reactive oxygen species (ROS), and HA synthase (HAS) [30]. When HA is degraded, the positive feedback of its fragment induces the production of various inflammatory intermediates and effectors such as free radicals, cytokines, chemokines, and enzymes that prime and perpetuate the inflammatory response, and consequently, tissue injury, as in the skin [31].

Mitogen-activated protein kinase 5 (*MEK5*) is the upstream activator of extracellular signal-regulated kinase 5 (*ERK5*) in many epithelial cells [32]. In vitro and in vivo investigations have reported that *MEK5*/*ERK5* pathway activation is frequently involved in inflammatory and oxidative responses [33]. *MEK5* promotes inflammatory responses to microbial stimuli in human endothelial and immune cells [34]. Numerous studies have reported that the *ERK5* protein controls the expression of a specific subset of inflammatory mediators in mouse epidermis and keratinocytes, triggering the recruitment of inflammatory cells necessary for skin carcinogenesis [35]. *ERK5* expression is increased in proliferative hypertrophic and mature scars [36]. In addition, some researchers have suggested variations in the distribution of HA in human skin and mature, hypertrophic, and keloid scars [37]. The level of HA is critical to the formation of scar tissue and the production of inflammation in keloid lesions [9]. Subsequently, we aimed to assess the potential role of the *MEK5*/*ERK5* pathway in HA degradation mechanisms.

**Figure 1:**
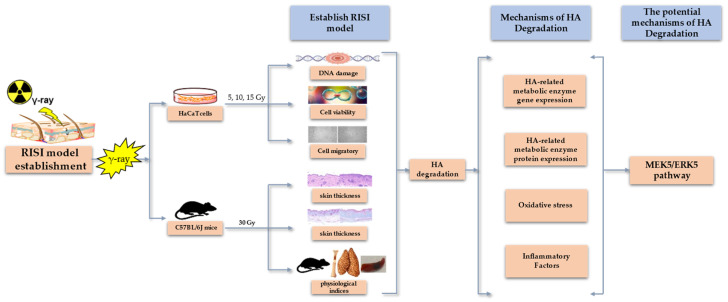
Graphical model of the effects of radiation-induced skin injury on hyaluronan degradation and its mechanisms.

In conclusion, RISI is a challenging problem owing to its complex pathogenesis, long course, lack of diagnostic gold standards, absence of specific drugs, and unclear mechanisms of occurrence [38]. Despite the crucial role of HA in skin function, studies investigating the relationship between HA and the RISI mechanism are scarce. Therefore, this study aimed to explore the impact of radiation on HA degradation in the skin and investigate the biological mechanisms underlying RISI and HA content changes (see Figure 1 below). These results may provide new insights into the occurrence and potential therapeutic targets of clinical RISI.

## 2. Results

### 2.1. Modeling of γ-Ray Irradiation Injury in HaCaT Cells

#### 2.1.1. γ-Ray Irradiation Enhanced HaCaT Cell DNA Damage

**Figure 2:**
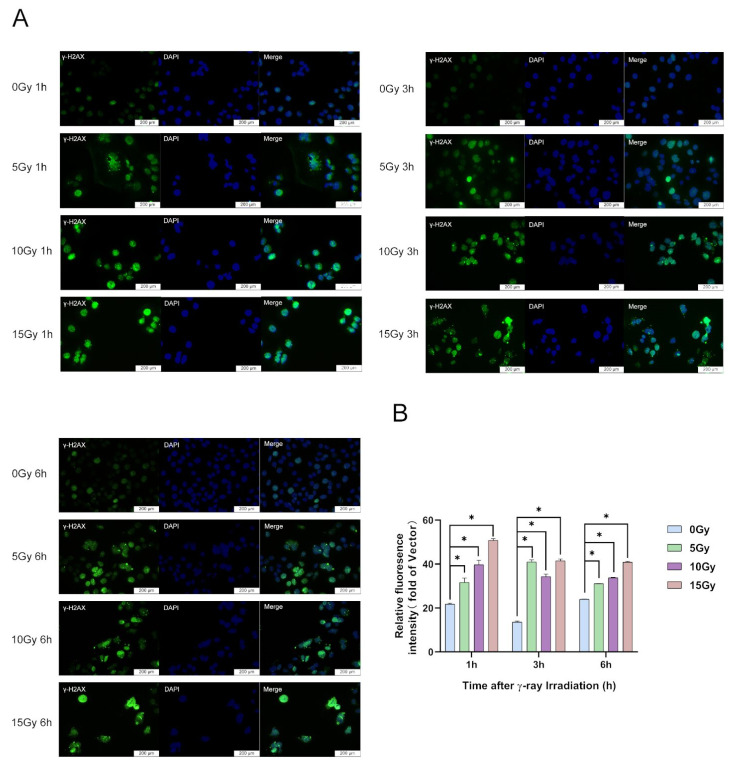
γ-ray irradiation-induced DNA damage of human keratinocyte (HaCaT) cells. (**A**,**B**) Representative images (**A**) and summary data (**B**) showing the degree of DNA damage 1, 3, and 6 h after γ-ray irradiation for HaCaT cells derived from unexposed (0 Gy) or exposed (5, 10, and 15 Gy) cells. Data are shown as the mean ± SEM (*n* = 3) of three independent experiments, * *p* < 0.05, which were analyzed using two-way analysis of variance followed by Tukey’s multiple comparisons test.

Based on the research of Wang (0, 2.5, 5, 10, 20 and 40 Gy; 6, 24, 48, 72 and 96 h) [39] and Lee et al. (0, 5 and 10 Gy) [17] and previous studies in our laboratory, in this study, we chose three irradiation doses—low (5 Gy), medium (10 Gy), and high (15 Gy)—to analyze the extent of DNA damage using a γ-H2AX immunofluorescence assay to determine the appropriate model for irradiation-injured cells. The results showed that γ-H2AX protein was substantially upregulated in HaCaT cells at 1, 3, and 6 h after 5, 10, and 15 Gy irradiation exposure, respectively (Figure 2A,B). At 1 and 6 h after irradiation, with increasing irradiation doses, γ-H2AX protein expression dose-dependently increased (Figure 2A,B). However, with the prolongation of the irradiation time, the protein expression of γ-H2AX was gradually downregulated (Figure 2A,B). When the irradiation dose was 5 Gy, the most significant increase in γ-H2AX protein expression was observed at 3 h (Figure 2A,B). In this study, we demonstrated that irradiation induces DNA damage. DNA damage in HaCaT cells occurs within a short time after irradiation (Figure 2A,B).

#### 2.1.2. γ-Ray Irradiation Reduced HaCaT Cell Viability

**Figure 3:**
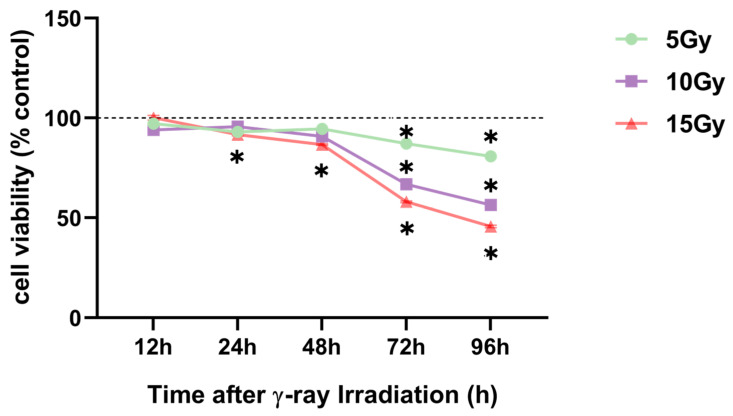
γ-ray irradiation-induced inhibition of human keratinocyte (HaCaT) cell proliferation. Summary data showing HaCaT proliferation as assessed using CCK-8 assays 12, 24, 48, 72, and 96 h after HaCaT cells were unexposed (0 Gy) and exposed (5, 10, and 15 Gy) to γ-ray irradiation. Data are shown as the mean ± SEM (*n* = 30) of three independent experiments, * *p* < 0.05, which were analyzed using two-way analysis of variance followed by Tukey’s multiple comparisons test.

The appropriate irradiation dose was selected before research was conducted on changes in HA content and degradation mechanisms in HaCaT cells. HaCaT cells were treated with γ-ray irradiation, with doses ranging from 0 to 15 Gy. The CCK-8 assay was performed to analyze cell viability. The results revealed a significant and dose-dependent reduced cell viability at 72 and 96 h after 5, 10, and 15 Gy irradiation exposure (Figure 3). In addition, the viability of HaCaT cells was similarly reduced by nearly 50% after 96 h of 10 Gy irradiation compared with that after 72 h of 15 Gy exposure (Figure 3). Furthermore, we conducted a wound-healing assay with 10 and 15 Gy exposure to select the appropriate radiation dose to determine the irradiation dose.

#### 2.1.3. γ-Ray Irradiation Reduced HaCaT Cell Migratory Ability and Level of Fibrosis

**Figure 4:**
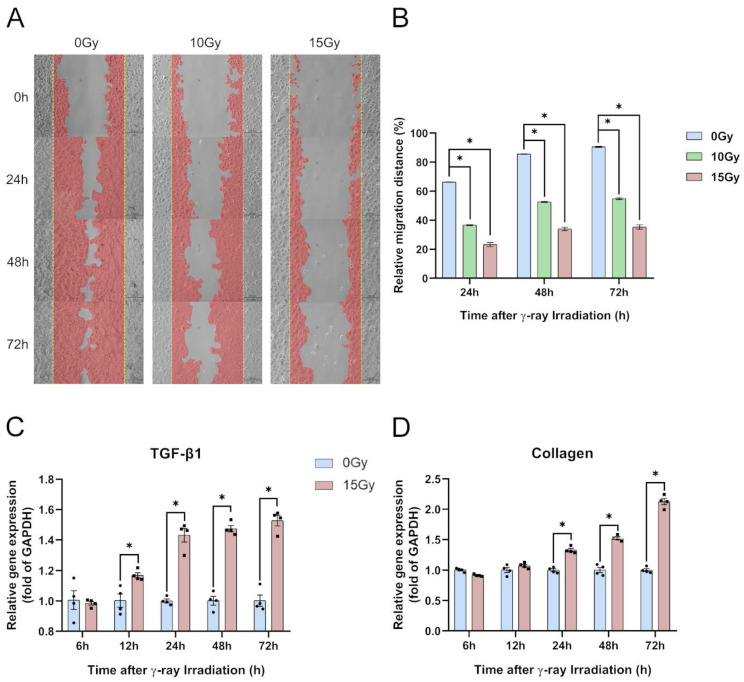
γ-ray irradiation-induced inhibition of cell migration and enhancement of the level of fibrosis of human keratinocyte (HaCaT) cells. (**A**,**B**) Representative images (**A**) and summary data (**B**) showing the degrees of migratory ability 24, 48, and 72 h following γ-ray irradiation for HaCaT cells derived from unexposed (0 Gy) or exposed (10 and 15 Gy) cells. (**C**,**D**) Gene expression levels of TGF-β1 and collagen as assessed using qRT-PCR assays 6, 12, 24, 48, and 72 h after HaCaT cells were unexposed (0 Gy) and exposed (15 Gy) to γ-ray irradiation. Data are shown as the mean ± SEM (*n* = 3) of three independent experiments, * *p* < 0.05, which were analyzed using two-way analysis of variance followed by Dunnett’s multiple comparisons test. The circular points refer to the data unexposed and square points refer to the data exposed (15 Gy) from multiple repeated experiments.

To simultaneously determine the cell proliferative and migratory abilities of HaCaT cells, we performed a wound-healing assay to monitor the specimens at 0, 24, 48, and 72 h (Figure 4A). All specimens were irradiated with 0, 5, 10, and 15 Gy, which revealed significantly reduced wound-healing ability. Reductions ranged from 33% to 51% within 48 h and 35% to 55% within 72 h after scratching (Figure 4B). The assay showed that the proliferative and migratory abilities of the HaCaT cells were dose-dependent (Figure 4A,B). Based on the experimental results on the migratory ability of HaCaT cells, we analyzed the gene expression of TGF-β1 and collagen in HaCaT cells after 15 Gy irradiation. We found that the TGF-β1 gene expression was significantly upregulated at 24, 48, and 72 h and the expression of collagen was significantly upregulated at 48 and 72 h after irradiation (Figure 4C,D). According to the wound-healing assay and level of fibrosis, we selected an irradiation dose of 15 Gy based on the results of the previous experiments to conduct the ensuing mechanism studies.

### 2.2. Impact of γ-Ray Irradiation on HA Degradation in HaCaT Cells

**Figure 5:**
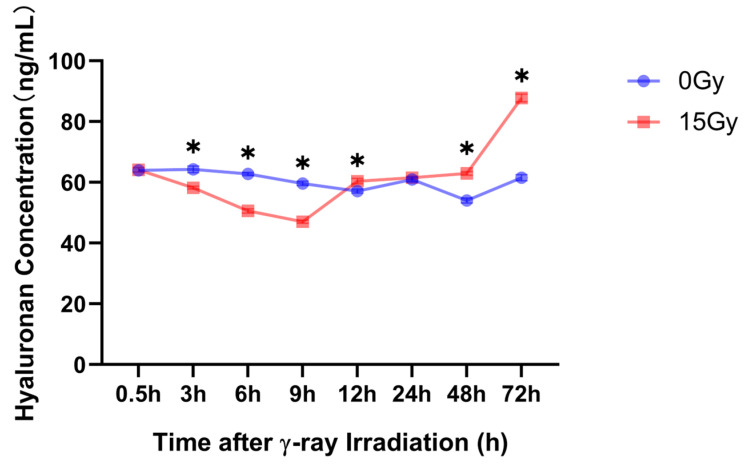
γ-ray irradiation affected the cell supernatant of the HA concentration in human keratinocyte (HaCaT) cells. Summary data showing the HA concentration as assessed using ELISA 0.5, 3, 6, 9, 12, 24, 48, and 72 h after HaCaT cells were unexposed (0 Gy) and exposed (15 Gy) to γ-ray irradiation. Data are shown as the mean ± SEM (*n* = 6) of three independent experiments, * *p* < 0.05, which were analyzed using two-way analysis of variance followed by Sidak’s multiple comparisons test.

The skin contains most of the body’s high-molecular-weight HA, which has numerous functions in the skin [40]. Herein, we used an enzyme-linked immunosorbent assay (ELISA) to detect the low-molecular-weight HA concentrations at 0.5, 3, 6, 9, 12, 24, 48, and 72 h after 15 Gy irradiation. The results revealed that with 12 h as the threshold after 15 Gy irradiation, the HA concentration in the supernatant of the cells significantly decreased within 12 h after radiation and was significantly upregulated after 12 h (Figure 5).

### 2.3. Mechanisms of γ-Ray Irradiation on HA Degradation in HaCaT Cells

Based on the theoretical basis of the mechanism of HA degradation related to HA-related enzymes, oxidative stress, and inflammatory response, and the results of previous experiments on the correlation between ionizing radiation and the content of low-molecular-weight HA, changes in HA-related metabolic enzyme expression levels, ROS production, and inflammatory factors were investigated following 15 Gy irradiation.

#### 2.3.1. Effect of γ-Ray Irradiation on Gene Expression of HA-Related Metabolic Enzymes in HaCaT Cells

**Figure 6:**
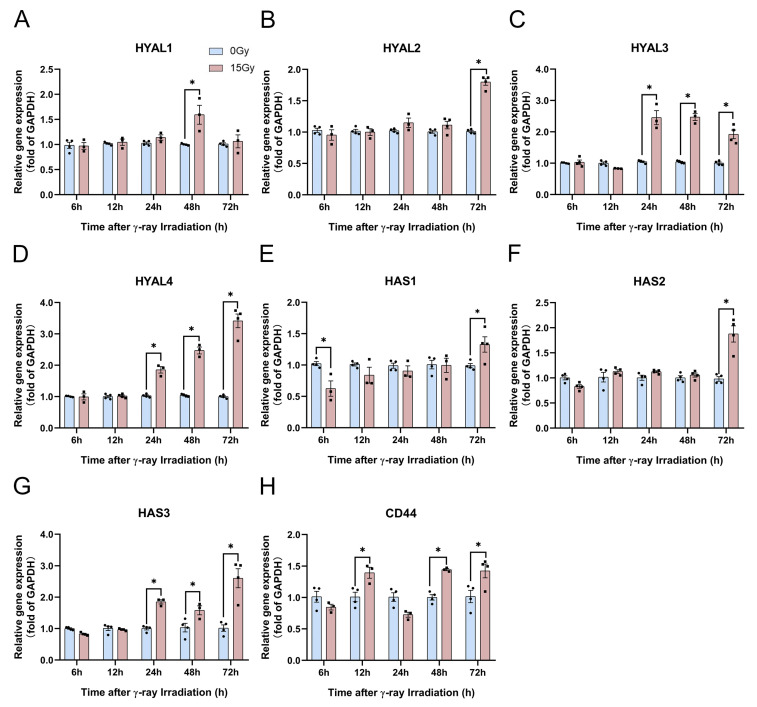
γ-ray irradiation affected the gene expression of HA-related metabolic enzymes in human keratinocyte (HaCaT) cells. (**A**–**H**) Gene expression levels of *HYAL1*, *HYAL2*, *HYAL3*, *HYAL4*, *HAS1*, *HAS2*, *HAS3*, and *CD44* as assessed using qRT-PCR assays 6, 12, 24, 48, and 72 h after HaCaT cells were unexposed (0 Gy) and exposed (15 Gy) to γ-ray irradiation. Data are shown as the mean ± SEM (*n* = 4) of three independent experiments, * *p* < 0.05, which were analyzed using two-way analysis of variance followed by Sidak’s multiple comparisons test. The circular points refer to the data unexposed and square points refer to the data exposed (15 Gy) from multiple repeated experiments.

Based on this research theory, we analyzed the gene expression of HA synthesis and degradation enzymes at 6, 12, 24, 48, and 72 h following 15 Gy irradiation using real-time quantitative polymerase chain reaction (PCR). The results revealed no significant effect on the expression of various HA-related metabolic enzyme genes within 12 h following irradiation. Only *HAS1* exhibited a significant inhibitory effect (Figure 6A–G). However, *HYAL3*, *HYAL4*, and *HAS3* were significantly upregulated after 24 h of irradiation (Figure 6C,D,G). *HAS2* and *HYAL2* are reportedly the main enzymes that play a role in HA synthesis and degradation in the human body [41]. Herein, *HAS2* and *HYAL2* gene expression levels were significantly enhanced at 72 h after 15 Gy irradiation (Figure 6B,F). Herein, the *CD44* gene expression levels were nonsignificant at 6 and 24 h but were significantly enhanced at 12, 48, and 72 h (Figure 6H). These results indicate that HA-related metabolic enzymes and their receptors were significantly enhanced at 48 h and in the late stages of irradiation exposure.

#### 2.3.2. Effect of γ-Ray Irradiation on Protein Expression of HA-Related Metabolic Enzymes in HaCaT Cells

**Figure 7:**
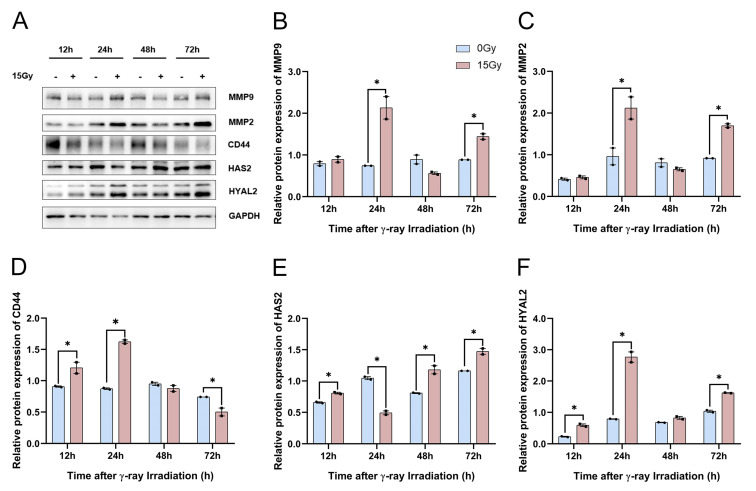
γ-ray irradiation affected the protein expression of HA-related metabolic enzymes in human keratinocyte (HaCaT) cells. (**A**–**F**) Representative images (**A**) and summary data (**B**–**F**) showing *MMP-9*, *MMP-2*, *CD44*, *HAS2*, and *HYAL2* protein expression levels in HaCaT cells as assessed using Western blotting assays 6, 12, 24, 48, and 72 h after HaCaT cells were unexposed (0 Gy) and exposed (15 Gy) to γ-ray irradiation. Data are shown as the mean ± SEM (*n* = 3) of three independent experiments, * *p* < 0.05, which were analyzed using two-way analysis of variance followed by Sidak’s multiple comparisons test. The circular points refer to the data unexposed and square points refer to the data exposed (15 Gy) from multiple repeated experiments.

In our previous study, we performed qRT-PCR to reveal that the transcript expression of HA-related metabolic enzymes increased significantly in HaCaT cells following γ-ray irradiation. Herein, we needed to confirm whether HA-related metabolic enzymes’ protein expression was significantly upregulated. In addition, the Western blotting results showed that *MMP-2*, *MMP-9*, and *HYAL2* were upregulated significantly at 24 h and 72 h after γ-ray irradiation (Figure 7A–C,F). We found that the *HAS2* protein expression was significantly reduced at 24 h, and after 48 h of 15 Gy irradiation, the expression of *HAS2* protein began to significantly regress with the prolongation of time (Figure 7A,E). The results demonstrated that *CD44* protein expression was significantly suppressed with increasing time after irradiation (Figure 7D). Together, these results suggested that the protein expression of *MMP-2*, *MMP-9*, *HAS2*, and *HYAL2* can be significantly upregulated, and that the protein expression of *CD44* can be significantly reduced after irradiation.

#### 2.3.3. Effect of γ-Ray Irradiation on ROS Production in HaCaT Cells

**Figure 8:**
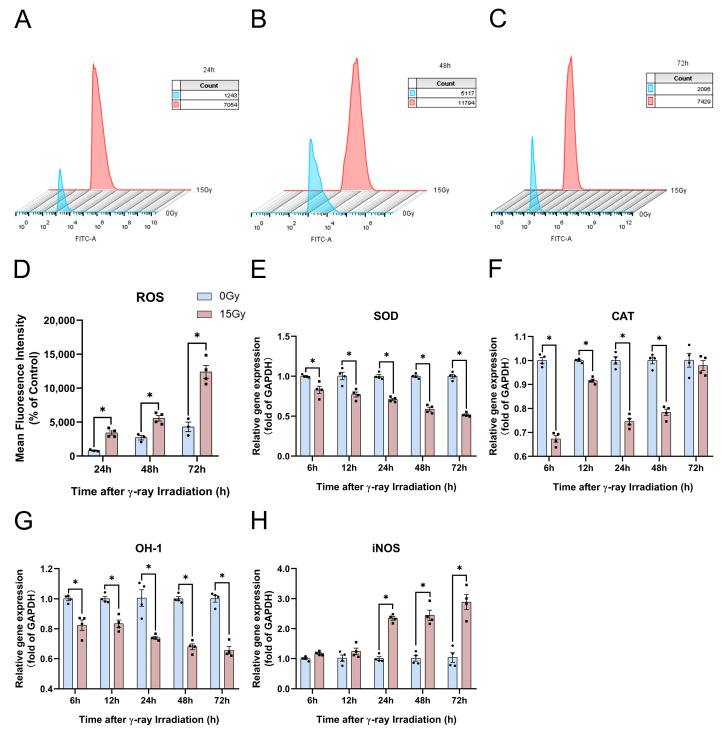
γ-ray irradiation affected the ROS content production in human keratinocyte (HaCaT) cells. (**A**–**D**) Representative images (**A**–**C**) and summary data (**D**) showing the ROS content production as assessed using flow cytometer assays 24, 48, and 72 h after HaCaT cells were unexposed (0 Gy) and exposed (15 Gy) to γ-ray irradiation. (**E**–**H**) Gene expression levels of SOD, CAT, OH-1 and iNOS as assessed using qRT-PCR assays 6, 12, 24, 48, and 72 h after HaCaT cells were unexposed (0 Gy) and exposed (15 Gy) to γ-ray irradiation. Data are shown as the mean ± SEM (*n* = 4) of three independent experiments, * *p* < 0.05, which were analyzed using two-way analysis of variance followed by Sidak’s multiple comparisons test. The circular points refer to the data unexposed and square points refer to the data exposed (15 Gy) from multiple repeated experiments.

To explore the ROS content production in HaCaT cells, we used the fluorescent probe DCFH-DA to stain intracellular ROS to observe the ROS content at 24, 48, and 72 h after 15 Gy irradiation (Figure 8A–C). We found that the ROS content was significantly upregulated at 24, 48, and 72 h after irradiation, and the ROS production increased with a certain irradiation dose and time dependence (Figure 8D).

Then, we further investigated the potential mechanisms of ROS production. We found that sodium dismutase (SOD) and OH-1 gene expression were significantly suppressed at 6, 12, 24, 48, and 72 h after irradiation (Figure 8E,G). SOD and OH-1 expression were extremely sensitive to irradiation, showing dose- and time-dependent irradiation (Figure 8E,G). The catalase (CAT) content decreased significantly at 6, 12, 24, and 48 h, and the CAT content gene expression was upregulated after irradiation (Figure 8F). At 24, 48, and 72 h after irradiation, ROS production was significantly upregulated, and the expression of antioxidant genes such as CAT, OH-1, and SOD was significantly inhibited. And iNOS gene expression was significantly increased, which increased NO synthesis and thus aggravated oxidative stress injury (Figure 8D–H). These results indicate that irradiation can induce significant ROS production and significantly upregulate the genes involved in the oxidative stress expression of OH-1, CAT, and SOD.

#### 2.3.4. Potential Mechanism Underlying the Effect of γ-Ray Irradiation on the *MEK5*/*ERK5* Pathway and Inflammatory Factors Involved in HA Degradation in HaCaT Cells

**Figure 9:**
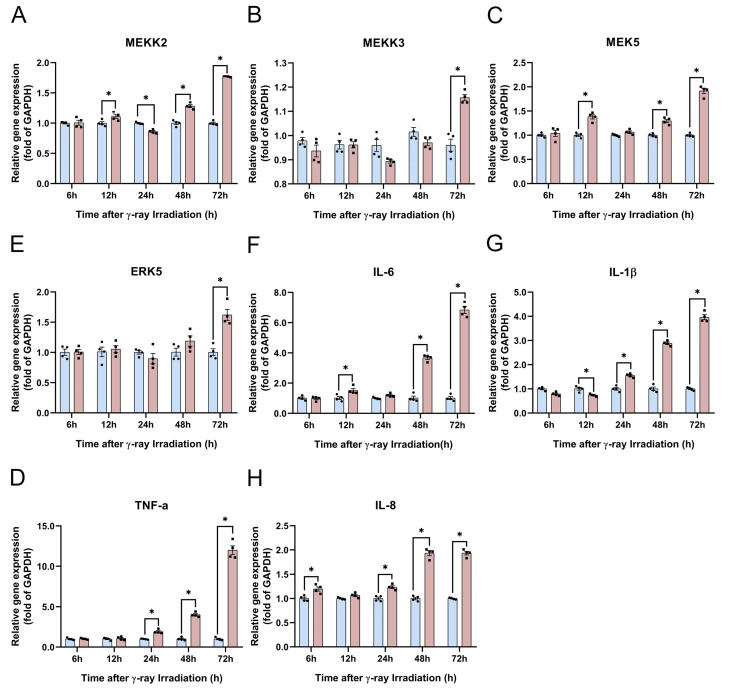
γ-ray irradiation affected the gene expression of inflammatory factors in human keratinocyte (HaCaT) cells. (**A**–**H**) Gene expression levels of *MEKK2*, *MEKK3*, *MEK5*, *ERK5*, *IL-1β*, *IL-6*, *TNF-α*, and *IL-8* as assessed using qRT-PCR assays 6, 12, 24, 48, and 72 h after HaCaT cells were unexposed (0 Gy) and exposed (15 Gy) to γ-ray irradiation. Data are shown as the mean ± SEM (*n* = 4) of three independent experiments, * *p* < 0.05, which were analyzed using two-way analysis of variance followed by Sidak’s multiple comparisons test. The circular points refer to the data unexposed and square points refer to the data exposed (15 Gy) from multiple repeated experiments.

In this section, we hypothesize that the *MEK5*/*ERK5* pathway may be subtly involved in the inflammation response and HA degradation. We used qRT-PCR to detect the gene expression of the *MEK5*/*ERK5* pathway and inflammatory factors of *MEKK2/MEKK3*, *MEK5*, *ERK5*, *IL-1β*, *IL-6*, *TNF-α*, and *IL-8*. We found that the gene expression of *MEKK2* was first significantly enhanced at 12 h, whereas *MEEK3* gene expression was increased at 72 h of irradiation (Figure 9A,B). The *MEK5* gene expression results showed that the *MEKK2* gene was the main gene regulating *MEK5* in the RISI model (Figure 9A,C). The expression of *ERK5*, the most downstream gene, was significantly upregulated only after 3 d of radiation (Figure 9D). Compared with HaCaT cells unexposed to irradiation, the gene expression of *IL-1β*, *TNF-α*, and *IL-8* was significantly increased at 24, 48, and 72 h (Figure 9E,G,H). Following the results for *IL-8* gene expression, we found that irradiation can instantly stimulate HaCaT cells and that *IL-8* was significantly enhanced at 6 h (Figure 9H). The *IL-6* gene expression was significantly increased at 12, 48, and 72 h after irradiation (Figure 9F). An interesting phenomenon is that *IL-1β* was suppressed at 6 h and 12 h (Figure 9E). These results indicate that with increasing time after radiation, the expression of the *MEK5*/*ERK5* pathway is biologically relevant to inflammatory factors. However, the upregulation of inflammatory factors is not only a result of the *MEK5*/*ERK5* pathway but is also regulated by other factors in RISI mice.

### 2.4. Modeling of γ-Ray RISI in C57BL/6J Mice

#### 2.4.1. γ-Ray Irradiation Induced Changes in Mice’s Skin Histopathology

**Figure 10:**
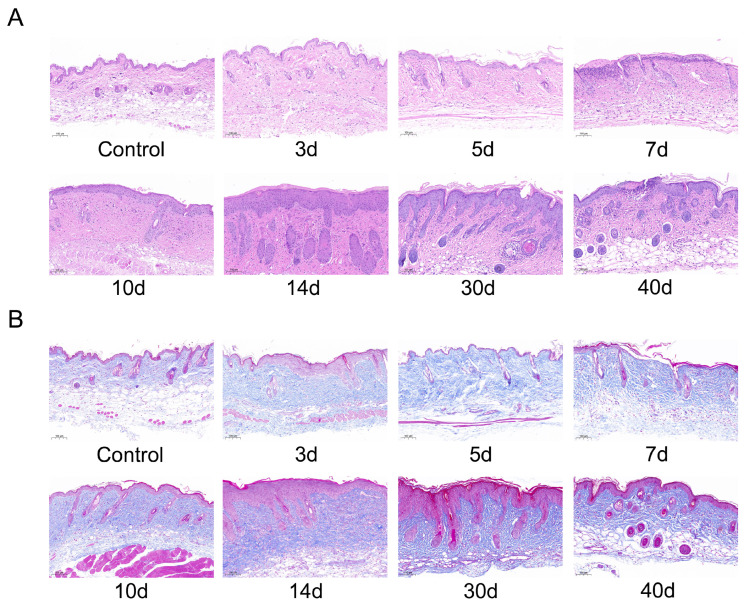

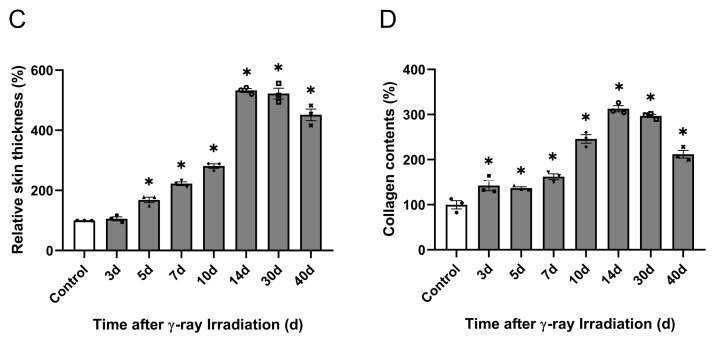
Effects of γ-ray irradiation on skin histopathology in C57BL/6J mice. (**A**,**B**) Representative images of HE and Masson staining in γ-ray-irradiated C57BL/6J mice’s skin tissues. (**C**,**D**) Quantification of the epidermal thickness (*n* = 3) and relative collagen area (%) (*n* = 3) in the experimental groups at different timepoints is shown for C57BL/6J mice that were unexposed (0 Gy) and exposed (30 Gy) after 3, 5, 7, 10, 14, 30, and 40 d of γ-ray irradiation. Data are shown as the mean ± SEM (*n* = 3) of three independent experiments, * *p* < 0.05, which were analyzed using one-way analysis of variance followed by Dunnett’s multiple comparisons test. The circular points refer to the data unexposed and square points refer to the data exposed (30 Gy) from multiple repeated experiments.

According to clinical high-dose-rate brachytherapy for malignant facial skin lesions with a cumulative radiation dose of 30 Gy [42], a preclinical model of RISI in mice has been proposed in the literature. Based on our knowledge of the effects of different doses of γ-rays on the skin, Mao et al.’s study on the dose of 30 Gy radiation was combined with our research platform in the laboratory to establish a RISI model in 30 Gy mice [43]. We chose 30 Gy irradiation to develop a hemi-body/partial body irradiation (PBI) model by exposing only the abdomen and lower extremities to study RISI and the effects of HA degradation and its mechanism. First, we used HE and Masson staining to detect epithelial thickening and collagen deposition in the skin tissue after 30 Gy irradiation. In this study, the enrichment of dermal tissue and progressive deposition of collagen were observed after irradiation. In the control group, the epidermis was intact, the structure was clear, the dermis was rich in collagen fibers, and the hair follicles and their accessory structures were normal. After 3, 5, 7, and 10 d of irradiation, the epidermis was mildly thickened, epidermal epithelial cells were mildly hyperplastic, and dermal hair follicles and their appendages showed no significant changes. After 30 d of irradiation, the epidermis thickened, the stratum corneum was hyperkeratinized, the number of epidermal epithelial cells increased, and the dermis had localized follicular necrosis and infiltration of inflammatory cells. After 40 d of irradiation, localized ulcers appeared in the epidermis and involved the dermis (Figure 10A). Masson staining showed that the dermal collagen fiber layer in the irradiation group was thicker than that in the normal dermal tissue, and relatively thin dermis and small hair follicles were observed in the control group (Figure 10B). Epidermal thickness increased and dermal collagen deposition increased after 14 d of irradiation compared with unirradiated controls. After 14 d of irradiation, the injured skin gradually recovered, epidermal thickness reduced, and dermal collagen area decreased (Figure 10C,D). These results indicate the successful modeling of localized RISI.

#### 2.4.2. γ-Ray Irradiation Altered the Physiological Indices of Mice

**Figure 11:**
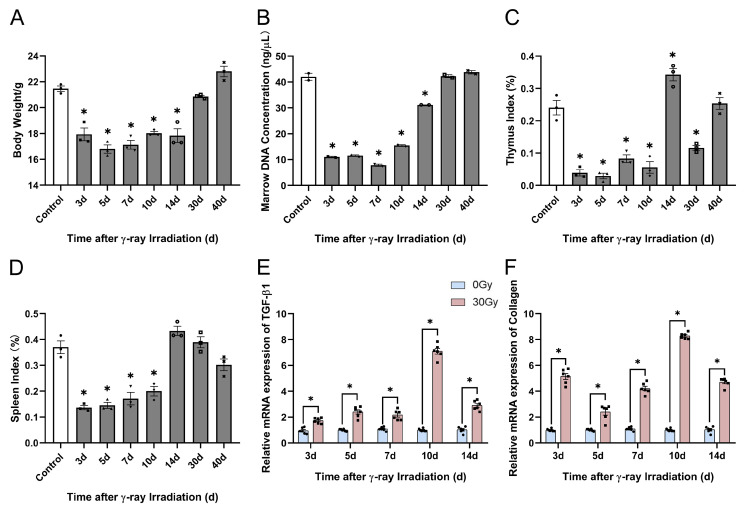
Effects of γ-ray irradiation on skin histopathology in C57BL/6J mice. (**A**–**D**) Physiological indices of body weight, marrow concentration, thymus index, spleen index, and the gene expression of TGF-β1 and collagen changed in C57BL/6J mice. (**E**,**F**) Gene expression levels of TGF-β1 and collagen as assessed using qRT-PCR assays 3, 5, 7, 10, and 14 d after C57BL/6J mice were unexposed (0 Gy) and exposed (30 Gy) to γ-ray irradiation. Data are shown as the mean ± SEM (*n* = 3) of three independent experiments, * *p* < 0.05, which were analyzed using one-way analysis of variance followed by Dunnett’s multiple comparisons test. The circular points refer to the data unexposed and square points refer to the data exposed (30 Gy) from multiple repeated experiments.

To further verify the modeling of RISI in C57BL/6J mice, we found that numerous studies have demonstrated that PBI can induce mouse body weight loss, severely affect the hematopoietic system, and scatter irradiation to body parts such as the spleen and sternum [44]. Therefore, we used physiological indices to prove the modeling of RISI in C57BL/6J mice. Herein, we found that the body weight and marrow DNA concentration of C57BL/6J mice significantly decreased within 14 d of irradiation. After 14 d of irradiation, the values of both the indices were upregulated and returned to normal (Figure 11A,B). Then, we detected the thymus and spleen indices, which significantly reduced within 10 d of irradiation. Furthermore, organ hypertrophy appeared in mice at 14 d of irradiation, followed by a decrease in the organ index after 14 d of irradiation (Figure 11C,D). Subsequently, we examined the gene expression levels of TGF-β1 and collagen in skin tissue and found that TGF-β1 and collagen genes were significantly upregulated 3, 5, 7, 10, and 14 d after 30 Gy irradiation (Figure 11E,F). These results indicate the successful modeling of RISI in C57BL/6J mice. Consequently, we selected a localized irradiation dose of 30 Gy to establish the RISI mouse model.

### 2.5. Impact of γ-Ray Irradiation on HA Degradation in C57BL/6J Mice

**Figure 12:**
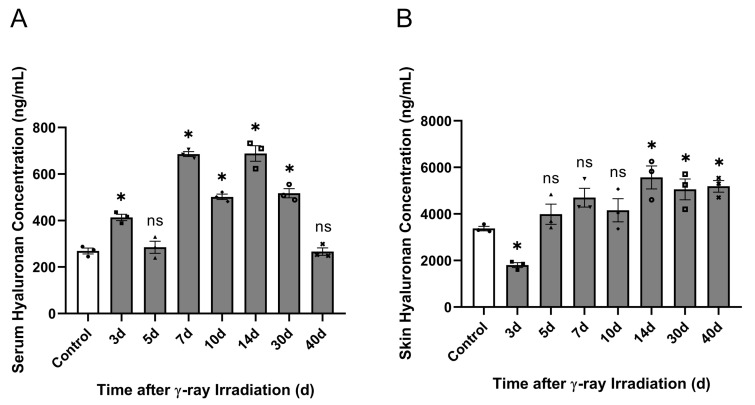
γ-ray irradiation affected the serum and skin HA content in C57BL/6J mice. (**A**,**B**) The levels of HA content in C57BL/6J mice that were unexposed (0 Gy) and exposed (15 Gy) after 3, 5, 7, 10, 14, 30, and 40 d of γ-ray irradiation assessed using ELISA. (**A**) Serum HA content. (**B**) Skin HA content. Data are shown as the mean ± SEM (*n* = 3) of three independent experiments, * *p* < 0.05, ns refers to not significant, which were analyzed using one-way analysis of variance followed by Dunnett’s multiple comparisons test. he circular points refer to the data unexposed and square points refer to the data exposed (30 Gy) from multiple repeated experiments.

In a previous study, we demonstrated that γ-ray irradiation can change HA concentration, resulting in a tendency for the concentration to decrease and then significantly increase in HaCaT cells. In vitro HaCaT cell experiments have suggested that irradiation injury affects HA degradation. Based on previous RISI-modeled C57BL/6J mice, we investigated the correlation between HA degradation and RISI in vivo. In the in vivo study, the low-molecular-weight HA content in the serum significantly increased at 3, 7, 10, 14, and 30 d after irradiation, reaching the highest level on day 14 (Figure 12A). Then, we detected the low-molecular-weight HA content in the skin, which significantly decreased at 3 d and significantly increased after 14 d of irradiation (Figure 12B). These results demonstrate that γ-ray irradiation increased the serum HA content during the middle stages and significantly increased skin tissue HA content during the later stages (Figure 12A,B).

### 2.6. Mechanisms Underlying the Effect of γ-Ray Irradiation on HA Degradation in C57BL/6J Mice

Given that previous results suggested that γ-ray irradiation affected serum and skin HA content, indicating that irradiation altered the HA degradation in the skin tissue of C57BL/6J mice, we investigated the mechanism of change in HA degradation. We analyzed HA degradation in the above HaCaT cell experiments, revealing that HA degradation is associated with increases in HA-related metabolic enzymes, ROS production, the *MEK5*/*ERK5* pathway, and inflammatory factors. In addition, we analyzed the mechanisms of HA degradation using qRT-PCR, Western blotting, SOD, and malondialdehyde (MDA) assays in RISI mice.

#### 2.6.1. Effect of γ-Ray Irradiation on HA-Related Metabolic Enzyme Gene Expression in C57BL/6J Mice

**Figure 13:**
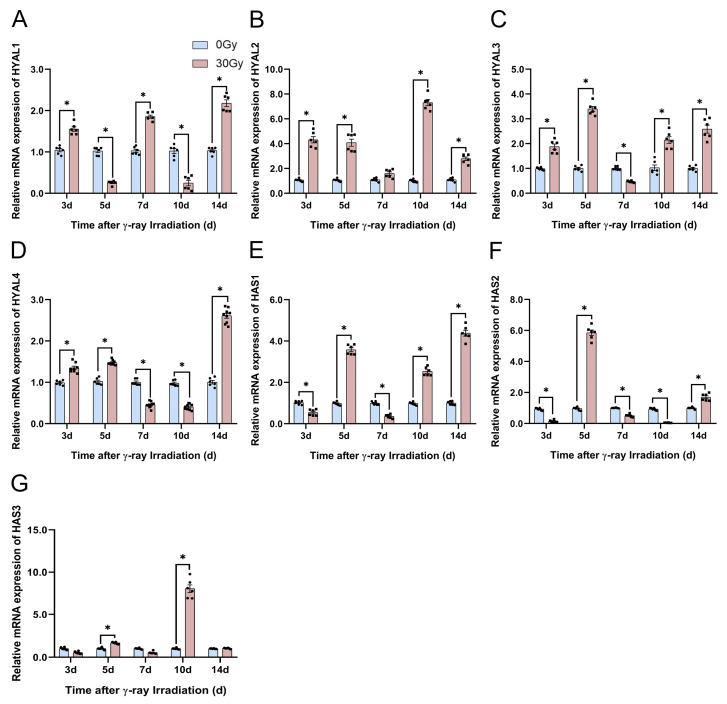
γ-ray irradiation affected the gene expression of HA-related metabolic enzymes in C57BL/6J mice. (**A**–**G**) Gene expression levels of *HYAL1*, *HYAL2*, *HYAL3*, *HYAL4*, *HAS1*, *HAS2*, and *HAS3* in C57BL/6J mice that were unexposed (0 Gy) and exposed (30 Gy) after 3, 5, 7, 10, and 14 d of γ-ray irradiation assessed using qRT-PCR assays. Data are shown as the mean ± SEM (*n* = 6) of three independent experiments, * *p* < 0.05, which were analyzed using two-way analysis of variance followed by Sidak’s multiple comparisons test. The circular points refer to the data unexposed and square points refer to the data exposed (30 Gy) from multiple repeated experiments.

First, we explored the HA-related metabolic enzyme gene expression using qRT-PCR on the skin tissue of C57BL/6J mice at 3, 5, 7, 10, and 14 d after irradiation. We found that the expression levels of HYAL1 significantly increased at 3, 7, and 14 d and significantly decreased at 5 and 10 d (Figure 13A). The HYAL3 and HYAL4 gene expression levels significantly increased at 3, 5, and 14 d and decreased at 3 and 7 d after irradiation (Figure 13C,D). The HAS1 and HAS3 gene expression levels significantly increased at 5 and 10 d and significantly decreased at 3 d (Figure 13E,G). We focused on the two most important genes, HYAL2 and HAS2, and discovered that the gene expression levels of HYAL2 were consistently upregulated following irradiation (Figure 13B) and the HAS2 gene was significantly repressed at all timepoints except at 5 and 14 d (Figure 13F). Based on the serum HA content, these HA-related metabolic enzyme gene expression results indicate that HA degradation was induced by the significant repression of HA-related synthase genes and significant activation of HA-related degrading enzyme genes.

#### 2.6.2. Effect of γ-Ray Irradiation on HA-Related Metabolic Enzyme Protein Expression in C57BL/6J Mice

**Figure 14:**
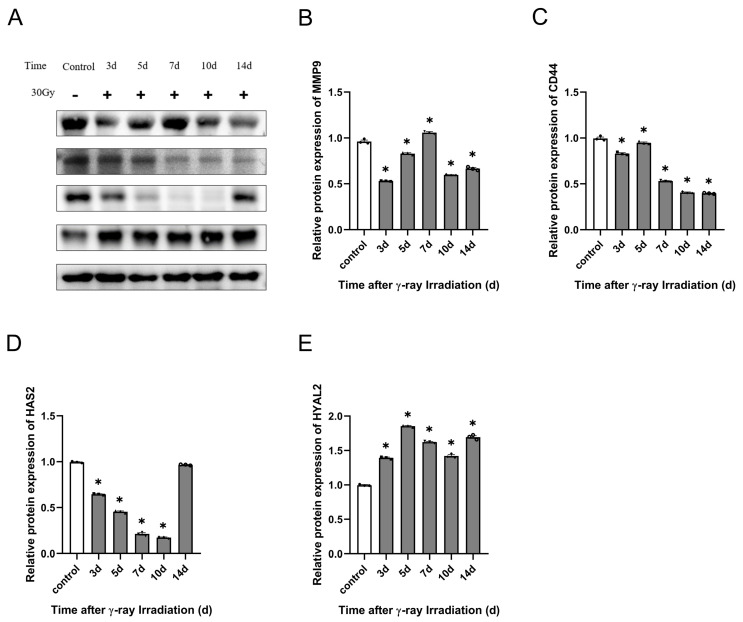
γ-ray irradiation affected the protein expression of HA-related metabolic enzymes in C57BL/6J mice. (**A**–**E**) Representative images (**A**) and summary data (**B**–**E**) showing MMP-9, CD44, HAS2, and HYAL2 protein expression levels in C57BL/6J mice that were unexposed (0 Gy) and exposed (30 Gy) after 3, 5, 7, 10, and 14 d of γ-ray irradiation assessed using Western blotting assays. Data are shown as the mean ± SEM (*n* = 3) of three independent experiments, * *p* < 0.05, which were analyzed using one-way analysis of variance followed by Dunnett’s multiple comparisons test. The circular points refer to the data unexposed and square points refer to the data exposed (30 Gy) from multiple repeated experiments.

From the foregoing study with qRT-PCR assays, it is clear that γ-ray irradiation can significantly affect the gene expression of HYAL2 and HAS2. We know that HYAL2, CD44, and HAS2 are important for HA degradation’s effect on HA concentration. Thus, we needed to detect the HA-related metabolic enzyme protein expression using a Western blotting assay after γ-ray irradiation. We discovered that CD44 protein expression decreased significantly at 3, 7, 10, and 14 d. Also, irradiation significantly stimulated CD44 protein enhancement at 5 d (Figure 14C). HAS2 is an essential HA synthase that decreased significantly at 3, 5, 7, and 10 d, and HAS2 protein expression gradually reverted to normal levels at 40 d of irradiation (Figure 14D). Then, we also detected the HA-degrading enzyme protein expression of HYAL2. Irradiation upregulated HYAL2 significantly for 2 weeks after irradiation (Figure 14E). Finally, we analyzed the protein expression of MMP-9 and found that MMP-9 gradually upregulated within 1 week after irradiation, with the highest expression on the 7th day, and then it was gradually downregulated after 7 d of radiation, showing significant inhibitory activities (Figure 14B). These results suggest that HA-related metabolic enzymes affect low-molecular-weight HA concentration in RISI C57BL/6J mice.

#### 2.6.3. γ-Ray Irradiation Induced Oxidative Stress in C57BL/6J Mice

**Figure 15:**
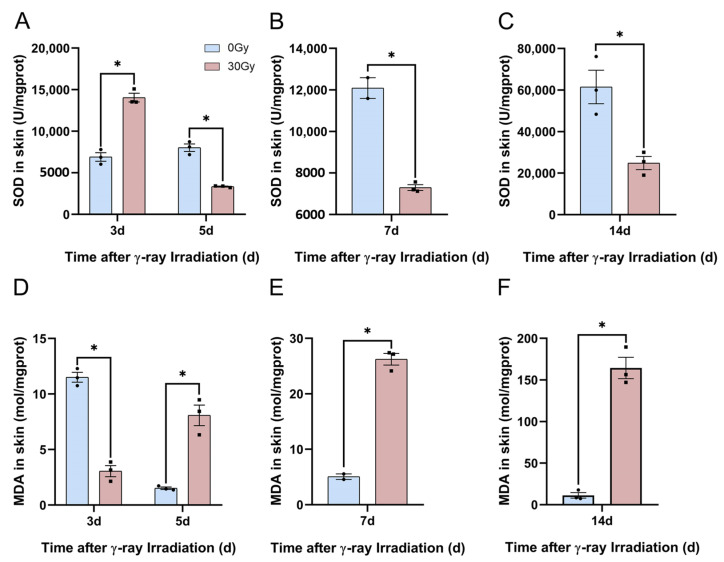
γ-ray irradiation affects the generation of oxidative stress in C57BL/6J mice. (**A**–**F**) Oxidative stress levels of SOD and MDA using SOD and MDA assays, respectively, to assess C57BL/6J mice that were unexposed (0 Gy) and exposed (30 Gy) after 7, 14, 30, and 40 d of γ-ray irradiation. Data are shown as the mean ± SEM (*n* = 3) of three independent experiments, * *p* < 0.05, which were analyzed using a two-tailed *t*-test analysis of variance followed by a paired comparisons test. The circular points refer to the data unexposed and square points refer to the data exposed (30 Gy) from multiple repeated experiments.

We have already summarized the mechanism of HA degradation. In in vitro cellular experiments, we demonstrated that low-molecular-weight HA content was first reduced and then increased, and ROS production was significantly enhanced following irradiation in HaCaT cells. For this section, we aimed to explore the indices of cutaneous oxidative stress to demonstrate the relation between HA degradation and oxidative stress. Consequently, we assessed the level of oxidative stress in skin tissue using SOD and MDA assays. The results revealed that SOD activity was significantly inhibited at 3, 5, 7, 10, and 14 d following irradiation (Figure 15A–C). The MDA concentration significantly increased after 3, 5, 7, 10, and 14 d of irradiation (Figure 15D–E). These results suggest that γ-ray irradiation induces oxidative stress in the skin. The generation of oxidative stress in RISI mice induces the degradation of high-molecular-weight HA to low-molecular-weight HA.

#### 2.6.4. Potential Mechanism Underlying the Effect of γ-Ray Irradiation on the *MEK5*/*ERK5* Pathway and Inflammatory Factors Involved in HA Degradation in C57BL/6J Mice

**Figure 16:**
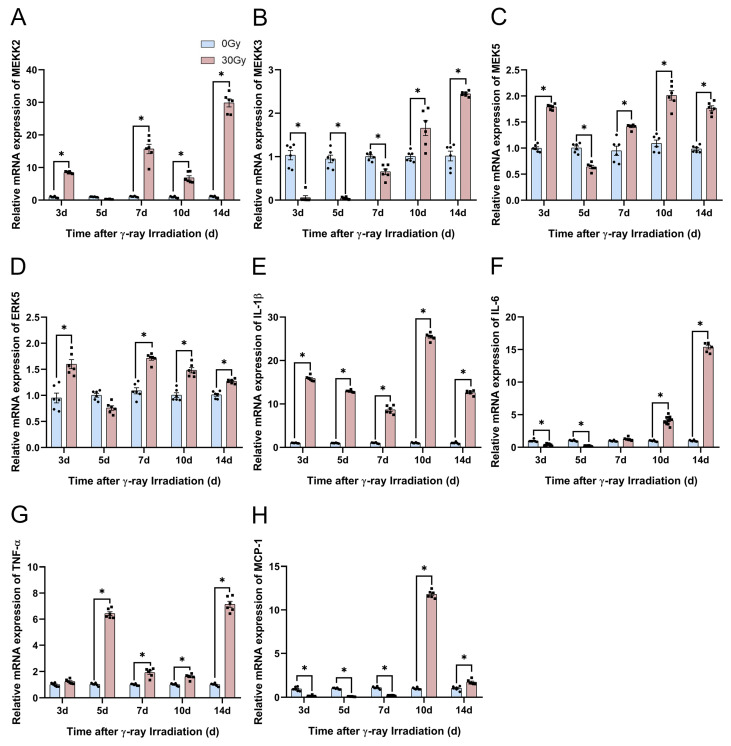
γ-ray irradiation affected the gene expression of inflammatory factors in C57BL/6J mice. (**A**–**H**) Gene expression levels of *MEKK2*, *MEKK3*, *MEK5*, *ERK5*, *IL-1β*, *IL-6*, *TNF-α*, and MCP-1 in C57BL/6J mice that were unexposed (0 Gy) and exposed (30 Gy) after 3, 5, 7, 10, and 14 d of γ-ray irradiation assessed using qRT-PCR assays. Data are shown as the mean ± SEM (*n* = 6) of three independent experiments, * *p* < 0.05, which were analyzed using two-way analysis of variance followed by Sidak’s multiple comparisons test. The circular points refer to the data unexposed and square points refer to the data exposed (30 Gy) from multiple repeated experiments.

We already demonstrated from in vitro cellular experiments that the *MEK5*/*ERK5* pathway relates to an inflammation response, and described that with the prolongation of irradiation, the expression of inflammatory factor genes and the HA concentration in the supernatant increased significantly. These in vitro cellular experiments indicated a correlation between HA degradation, the *MEK5*/*ERK5* pathway, and inflammatory factors. Therefore, we detected the expressions of the *MEK5*/*ERK5* pathway and inflammatory factors, including *MEKK2*, *MEKK3*, *MEK5*, *ERK5*, *IL-1β*, *IL-6*, *TNF-α*, and MCP-1 mRNA, in in vivo skin, and they were examined using a qRT-PCR assay. In the in vivo RISI model, the *MEKK2* gene was significantly activated at 3, 7, 10, and 14 d after irradiation, and the MEKK3 gene was significantly repressed within 7 d of irradiation and activated after 10 d of irradiation (Figure 16A,B). Within 7 d of irradiation, the gene expression of *MEK5* was mainly activated by its upstream gene, *MEKK2*, and then after 7 d of irradiation, it was coactivated by the *MEKK2* and *MEKK3* genes (Figure 16A–C). *MEK5* acts as the unique upstream activation gene for *ERK5*, and significantly activated the *ERK5* gene at 3, 7, 10, and 14 d after irradiation (Figure 16D). Subsequently, we revealed that *IL-1β* gene expression upregulated significantly within 2 weeks of irradiation (Figure 16E). The expression of the *IL-6* gene was significantly suppressed 5 d after irradiation and then upregulated 7 d after irradiation as the duration of irradiation was prolonged (Figure 16F). The gene expression of *TNF-α* increased significantly at 5, 7, 10, and 14 d of irradiation (Figure 16G). Finally, we assayed the MCP-1 gene expression, which was significantly suppressed within 7 d after irradiation, and continuously upregulated after 10 d of irradiation (Figure 16H). In conclusion, changes in the *MEK5*/*ERK5* pathway and inflammatory factors’ gene expression are consistent with cutaneous HA degradation in vivo.

## 3. Discussion

RISI is characterized by a long incubation period, being a recurring condition, a difficult reversal, having no effective therapeutic drugs, having no diagnostic gold standard, and an unclear occurrence mechanism, thereby rendering its clinical diagnosis and treatment challenging. Currently, there are numerous limitations to the studies of RISI, including the complexity of the structural composition of the skin, which poses a challenge to studying the extent and mechanism of skin tissue injury. Following the rapid development of genomics and proteomics, studying the mechanisms underlying RISI is restricted to these two groups, and there remains a significant gap in the research regarding the mechanisms underlying slowly developing glycobiology [45]. The search for a new target for the clinical treatment of RISI under the premise of accurate noninvasive diagnosis, precise mechanisms, and targeted therapy has thus become an urgent challenge to be solved.

HA plays a vital role in the skin. Numerous studies have reported that HA in the skin is responsible for the skin’s hydration and viscoelasticity and protection from free radicals, especially by sequestering ROS generated via ultraviolet (UV) radiation [46,47]. Several studies have proven that high-molecular-weight HA can immobilize water in the tissue, changing dermal volume and compressibility, and can influence cell proliferation, differentiation, tissue repair, regeneration, angiogenesis, and immune response [48,49,50,51]. Studies have shown that high-molecular-weight HA protects the skin from free irradiation injury, particularly via the sequestration of ROS generated through UV radiation, which is of substantial importance, whereas low-molecular-weight HA is a potent proinflammatory molecule [47]. To date, many researchers have concluded that the mechanism of HA degradation comprises depolymerization via specific endoglycosidases of hyaluronidases or nonspecific degradation via oxidative damage due to ROS and reactive nitrogen species [26,52]. In addition, highly polymerized HA is broken down into small components in the context of inflammatory pathologies, indicating that inflammatory factors can degrade HA [53]. Herein, in vitro cellular experiments and in vivo animal experiments proved that the HA content in skin tissues decreased and then increased following irradiation. The results illustrate that γ-ray irradiation can affect HA degradation and synthesis and thus the metabolism of HA. Thus, HA degradation is extremely important for RISI development and can be a potential treatment target. Furthermore, the main mechanisms underlying HA degradation in the RISI mouse model include HA-related metabolic enzymes, oxidative stress, and inflammatory factors. We demonstrated that γ-ray irradiation can significantly upregulate the expression of HA-related metabolic enzymes, oxidative stress, and inflammatory factors. These findings are consistent with the three currently reported mechanisms of HA degradation. Low-molecular-weight HA content significantly increased in the serum from 7 to 30 d following irradiation. However, low-molecular-weight HA content significantly decreased in the skin tissue at 3 d and then significantly increased after 14 d of irradiation.

We already knew from previous studies that inflammatory factors can break down highly polymerized HA into small fragments, and the *MEK5*/*ERK5* pathway can promote inflammatory factor secretion [35]. Given the crucial contribution of the *MEK5*/*ERK5* pathway to oxidative stress and inflammatory responses, we investigated the potential mechanisms of the *MEK5*/*ERK5* pathway in HA degradation after radiation exposure. γ-ray irradiation altered the gene expression of the *MEK5*/*ERK5* pathway in the skin tissues of RISI C57BL/6J mice. Alterations in the gene expression of the *MEK5*/*ERK5* pathway and HA content revealed a tendency to decrease and then increase following irradiation. These results suggest that HA degradation is associated with the *MEK5*/*ERK5* pathway. In addition, the *MEK5*/*ERK5* pathway regulates HA degradation, possibly by regulating the secretion of inflammatory factors.

There is a lack of targeted drugs with good efficacy and low adverse effects for the clinical prevention and treatment of γ-ray irradiation-induced skin injury. Therefore, exploring the mechanisms of irradiation skin injury using human keratinocyte cell lines and C57BL/6J mice is crucial. Recently, the search for potential therapeutic targets has become an important direction of radiological research. The findings of this study reveal that HA degradation is central to the mechanism of RISI. Initially, we explored the mechanisms of potential HA degradation. Then, based on our understanding of RISI and HA mechanisms, we hypothesized whether it would be possible to treat RISI from the perspective of inhibiting HA degradation. Ultimately, this study’s findings provide a new potential target for preventing and treating RISI. However, our study only demonstrated a direct correlation between RISI and HA content alteration. We only conducted preliminary correlation studies to evaluate the potential HA degradation mechanism. We did not conduct further rescue experiments to explore the precise targets of the HA degradation mechanism. In a future study, we will evaluate these targets and provide further comments.

## 4. Materials and Methods

### 4.1. Animals and Cells

Male C57BL/6J mice (*n* = 56) were purchased from Beijing Weitong Lihua Experimental Animal Technology Co., Ltd. The mice were 6–8 weeks of age, weighing 18.0–21.0 g. The Animal Care and Ethics Committee at the Institute of Radiation Medicine approved all experimental procedures. The mice were kept in an animal room with a constant temperature of 20 ± 2 °C and allowed free access to food and water.

HaCaT cells were purchased from ATCC (CRL-2310) and maintained in DMEM/F12 growth medium (cat. no. C11330500BT, Gibco, Grand Island, NY, USA) with 10% fetal bovine serum (cat. no. SH30084.03, HyClone, Logan, UT, USA) and 1% streptomycin/penicillin (cat. no. 15140-122, Gibco, Grand Island, NY, USA). HaCaT cells were grown in a 5% CO_2_ atmosphere at 37 °C.

### 4.2. γ-Ray Irradiation

Mice were randomly divided into a control and an irradiation group. The irradiation group was exposed to ^60^Co γ-rays, and the dose was 30 Gy. The dose rate was 108.21 cGy/min. Control mice were placed under the same conditions but without irradiation.

The HaCaT cell experiments were performed with reference to the irradiation dose [18]. Then, HaCaT cells were grouped similarly to the grouping of mice and irradiated at doses of 5, 10, and 15 Gy. The dose rate was 71.31 cGy/min. HaCaT cells from the control group were placed under the same conditions but without irradiation.

### 4.3. ELISA of HA

The mouse serum and skin HA contents were determined using ELISA according to the manufacturer’s instructions (cat. no. DHYAL0, R&D Systems, Emeryville, CA, USA). The detectable limits of HA were 0.625 ng/mL, and each sample was read in duplicate and averaged before analysis.

### 4.4. Reverse Transcription Quantitative Real-Time PCR Assay

Total RNA was extracted from the skin tissue of the mice using TRIzol reagent (Thermo Fisher Scientific, Waltham, MA, USA). RNA extraction reagent (cat. no. RC112-01, Vazyme, Nanjing, China) was used to isolate total RNA from HaCaT cells. A NanoDrop2000 spectrophotometer (Thermo Fisher Scientific, Waltham, MA, USA) was used for RNA quality control. cDNA was synthesized according to the manufacturer’s protocol (cat. no. RC333-01, Vazyme, Nanjing, China). We used a PrimeScript RT Master Mix kit (cat. no. Q711-02, Vazyme, Nanjing, China) for reverse transcription and the SYBR Green I fluorescence method for quantification. The mRNA levels were normalized to that of GAPDH. The 2^−∆∆CT^ method was used to calculate relative gene expression levels. The primers used for qRT-PCR are summarized in Table S1.

### 4.5. Western Blotting

Total proteins from animals and cells were extracted using RIPA (cat. no. C1053, Applygen, Beijing, China) buffer with protease inhibitor cocktail (Roche). The concentrations were determined using a BCA kit (cat. no. P0010, Beyotime, Shanghai, China). Sodium dodecyl sulfate–polyacrylamide gel electrophoresis (7.5% gels, cat. no. PG111, EpiZyme, Shanghai, China) was performed to separate equal amounts of proteins. The proteins were transferred to polyvinylidene difluoride membranes and blocked using 5% bovine serum albumin at room temperature for 1 h. The following membranes were immersed into an antibody incubation box loaded with the corresponding primary antibody and incubated in a 4 °C refrigerator overnight: *MMP-9* (cat. no. ab283575, Abcam, England and Wales, UK, dilution ratio 1:1000), *MMP-2* (cat. no. ab235167, Abcam, England and Wales, UK, dilution ratio 1:2000), *CD44* (cat. no. ab243894, Abcam, England and Wales, UK, dilution ratio 1:3000), *HAS2* (cat. no. ab140671, Abcam, England and Wales, UK, dilution ratio 1:2000), and *HYAL2* (cat. no. ab68608, Abcam, England and Wales, UK, dilution ratio 1:1000). The next day, secondary goat IgG antibody (cat. no. ZB-5301 or ZB-5305, Zsbio, Melbourne, Australia, dilution ratio 1:5000) was incubated with the membranes for 1 h. The target protein bands were developed in the instrument of the gel imaging system (Tanon, Shanghai, China), and the development of the target protein bands was observed. Images (Figure 6A and Figure 13A) have been cropped for presentation.

### 4.6. Wound-Healing Assay

HaCaT cells were inoculated onto a six-well plate, with same cell density in each well. When the cells reached 80% confluence, they were scraped using a sterile pipette tip. The medium was changed, and the cells were cultured in a 37 °C 5% CO_2_ incubator. The images of the cells in the wells were obtained at 0, 24, 48, and 72 h after radiation exposure. ImageJ software (version 1.8.0, Bethesda, CA, USA) was used to analyze the area of traces and calculate the cell migration rate.

### 4.7. Cell Viability Assay

HaCaT cells were evenly seeded in a 96-well plate, achieving the density of 5000 cells/well. At six timepoints (0, 12, 24, 48, 72, and 96 h), we discarded the medium and then incubated the cells with 90 µL culture medium and 10 µL CCK-8 (cat. no. A311-01, Vazyme, Nanjing, China) in each well. After placing the 96-well plate in the incubator for 2 h, we detected the absorbance at a wavelength of 450 nm using a microplate reader (Molecular Devices, Shanghai, China).

### 4.8. Analysis of γ-H2AX

HaCaT cells were fixed, and γ-H2AX immunofluorescence staining was performed 1, 3, and 6 h after irradiation. We used the γ-H2AX assay to detect DNA damage (cat. no. C2035S, Beyotime, Shanghai, China). The slides were visualized and photographed with an anti-fluorescence quenching blocking solution containing DAPI to calculate the average number of γ-H2AX foci per cell.

### 4.9. Analysis of ROS

The fluorescent dye 2′,7′-dichlorofluorescin diacetate (H2DCF-DA, cat. no. S0033S, Beyotime, Shanghai, China) was used to measure the intracellular ROS content. Cells with an appropriate density were incubated with 5 μM H2DCF-DA for 20 min at 37 °C, and then H2DCF oxidation was detected using flow cytometry.

### 4.10. Hematoxylin and Eosin Staining Assay

We used a standard protocol with hematoxylin and eosin staining. The mice were executed, and the skin was quickly removed and fixed with 4% paraformaldehyde (cat. no. G1101, Servicebio, Wuhan, China) for 24 h. Then, the skin samples were trimmed, dehydrated, embedded, sectioned, stained, and sealed according to the pathological experimental test standard operation procedure. Finally, the samples were qualified for microscopy.

### 4.11. Masson Staining Assay

We used the Masson staining assay to detect collagen fibers in the skin after irradiation. Paraffin-embedded skin tissue sections were dewaxed and hydrated. First, we used hematoxylin to stain nuclei for 5 min, followed by washing with Tris-buffered saline for 10 min. Then, we added Masson staining solution (cat. no. G1006, Servicebio, Wuhan, China) for 5 min. Next, we immersed the sections in 1% glacial acetic acid solution (cat. no. A116166, Aladdin, Shanghai, China) for 5–10 s. Finally, we mounted the sections with neutral gum. Images were taken under an optical microscope. We randomly selected four fields from each image for analysis.

### 4.12. Statistical Analysis

All data are expressed as mean ± SEM. GraphPad Prism software (version 8.3.0.538, San Diego, CA, USA) was used for statistical analysis and preparing graphs. One-way and two-way analyses of variance, or a *t*-test to compare two groups, were used to evaluate the data, and a *p*-value < 0.05 was considered statistically significant.

## 5. Conclusions

In HaCaT cells and a RISI C57BL/6J mouse model, our study demonstrated that γ-ray irradiation inhibits the expression of low-molecular-weight HA in the pre-exposure stage and significantly upregulates HA expression in the postexposure stage of RISI. The mechanisms underlying γ-ray irradiation-induced HA degradation comprise four major categories: (I) direct degradation of HA by HA-related metabolic enzymes; (II) interruption of HA glycosidic bonds by ROS; (III) indirect degradation of HA by the upregulation of inflammatory factors; and (IV) the potential degradation mechanism of HA by the *MEK5*/*ERK5* pathway. These findings provide new insights into the mechanisms of RISI and offer a potential target for RISI drug development.

## Data Availability

Data will be made available by the corresponding author upon reasonable request.

## Supplementary Materials

The following supporting information can be downloaded at https://www.mdpi.com/article/10.3390/molecules28217449/s1: Table S1: qRT-PCR primers used in this study.
