# Supplementary material for: Effects of Radiation-Induced Skin Injury on Hyaluronan Degradation and Its Underlying Mechanisms
Source: Molecules. 2023 Nov 6;28(21):7449. doi: 10.3390/molecules28217449 (PMC10647323; doi:10.3390/molecules28217449)
Supplement: Supplementary file 1 [file molecules-28-07449-s001.zip › Table S1.pdf]

Table S1. qRT-PCR primers used in this study.

Target Gene	Primers	Sequence (5′ → 3′)
GAPDH（human）	Forward primer	GGAGCGAGATCCCTCCAAAAT
	Reverse primer	GGCTGTTGTCATACTTCTCATGG
GAPDH（mouse）	Forward primer	TGGCCTTCCGTGTTCCTAC
	Reverse primer	GAGTTGCTGTTGAAGTCGCA
HYAL1（human）	Forward primer	TATGGCCCAAGGCTTTAGGG
	Reverse primer	GCTACCACATCGAAGACACTGA
HYAL2（human）	Forward primer	GAGCACTACATTCGGACACAG
	Reverse primer	GATAACCGGCGATACACATCTT
HYAL3（human）	Forward primer	TGGCTGATGCAGGTTTCCAT
	Reverse primer	GCGTATCCTCCATCAGTGCA
HYAL4（human）	Forward primer	ACGAAGTCTTGAGGAACAATGAG
	Reverse primer	TTTCCAGACACCGATAGAAGGA
HYAL1（mouse）	Forward primer	ACCTGCTTCGCATCTCTACTC
	Reverse primer	GGTTGGATACCACGGAACCTC
HYAL2（mouse）	Forward primer	GCAGGACTAGGTCCCATCATC
	Reverse primer	TTCCATGCTACCACAAAGGGT
HYAL3（mouse）	Forward primer	TCTGTGGTATGGAATGTACCCT
	Reverse primer	TTTTGGCCGTGAAAATGTTGG
HYAL4（mouse）	Forward primer	ATGCAACTATTGCCTGAAGGAC
	Reverse primer	GGGCAGGTTTTAGGGATGAGAT
HAS1（human）	Forward primer	GAGCCTCTTCGCGTACCTG
	Reverse primer	CCTCCTGGTAGGCGGAGAT
HAS2（human）	Forward primer	CTCTTTTGGACTGTATGGTGCC
	Reverse primer	AGGGTAGGTTAGCCTTTTCACA
HAS3（human）	Forward primer	CAGCCTATGTGACGGGCTAC
	Reverse primer	CCTCCTGGTATGCGGCAAT
HAS1（mouse）	Forward primer	ATGAGGACCCCGCCACTTAT
	Reverse primer	CACCTGCGTGTTCTCACCAG
HAS2（mouse）	Forward primer	TGTGAGAGGTTTCTATGTGTCCT
	Reverse primer	ACCGTACAGTCCAAATGAGAAGT
HAS3（mouse）	Forward primer	CAATCGCCAGGAAGATACCTAC
	Reverse primer	GGAAATTGCTACGCCACACAA
MEKK2（human）	Forward primer	CCCCAGGTTACATTCCAGATGA
	Reverse primer	GCATTCGTGATTTTGGATAGCTC
MEKK2（mouse）	Forward primer	ATGCTCAGCTCTCAATAGTA
	Reverse primer	ATGCGTAACTATTCAAGCAT
MEKK3（human）	Forward primer	GGCGAATTATAGCGTTCAGCC
	Reverse primer	GGGACAACAGCAATATCCTAAGG
MEKK3（mouse）	Forward primer	ATGTGAAGCTTGGGGATTTTG
	Reverse primer	TGGTTAGACTCACTGGTCAGAGAC
MEK5（human）	Forward primer	TCTGCCTGAAGCAACAACTAC
	Reverse primer	GCCATGTATGTTCCGTTCCC
MEK5（mouse）	Forward primer	GCCGCTGCAGATATTTCCAA
	Reverse primer	CAGCCCGTGTATTCACCTTCA
ERK5（human）	Forward primer	CCTGAAGCCTACTGTGCCCTATG
	Reverse primer	CCGAAGCAGCTGGTACAGGAA
ERK5（mouse）	Forward primer	TGAACCCAGTGCCCGAAT
	Reverse primer	GGCGCAATCAGGCTCATC
IL-1β（human）	Forward primer	ATGATGGCTTATTACAGTGGCAA
	Reverse primer	GTCGGAGATTCGTAGCTGGA

Target Gene	Primers	Sequence (5′ → 3′)
IL-1β（human）	Forward primer	GAAATGCCACCTTTTGACAGTG
	Reverse primer	TGGATGCTCTCATCAGGACAG
IL-6（human）	Forward primer	ACTCACCTCTTCAGAACGAATTG
	Reverse primer	CCATCTTTGGAAGGTTCAGGTTG
IL-6（mouse）	Forward primer	GCCTTCTTGGGACTGATGCT
	Reverse primer	GGTCTGTTGGGAGTGGTATCC
TNF-a（human）	Forward primer	CCTCTCTCTAATCAGCCCTCTG
	Reverse primer	GAGGACCTGGGAGTAGATGAG
TNF-a（mouse）	Forward primer	GAGAAGGGGGACCAACTCAG
	Reverse primer	CTCCAAAGTAGACCTGCCCG
CD44（human）	Forward primer	GGAGGGCAGCACTGTTTTTG
	Reverse primer	GCCACAAAGGACTTGCCAAG
SOD（human）	Forward primer	GGTGGGCCAAAGGATGAAGAG
	Reverse primer	CCACAAGCCAAACGACTTCC
CAT（human）	Forward primer	TGGAGCTGGTAACCCAGTAGG
	Reverse primer	CCTTTGCCTTGGAGTATTTGGTA
OH-1（human）	Forward primer	GCTCAACATCCAGCTCTTTGAGG
	Reverse primer	GACAAAGTTCATGGCCCTGGGA
IL-8（human）	Forward primer	TAGCCAGGATCCACAAGTCC
	Reverse primer	GCTTCCACATGTCCTCACAA
MCP-1（mouse）	Forward primer	CAGCCAGATGCAATCAATGCC
	Reverse primer	TGGAATCCTGAACCCACTTCT
