# Supplementary material for: Effects of Radiation-Induced Skin Injury on Hyaluronan Degradation and Its Underlying Mechanisms
Source: Molecules. 2023 Nov 6;28(21):7449. doi: 10.3390/molecules28217449 (PMC10647323; doi:10.3390/molecules28217449)

HaCaT WB original image

MMP9

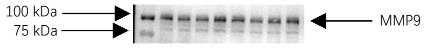

CD44

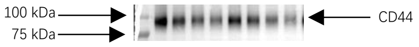

HAS2

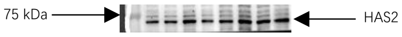

GAPDH

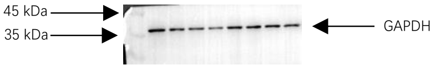

MMP2

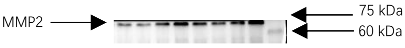

HYAL2

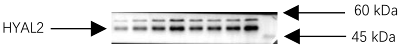

GAPDH

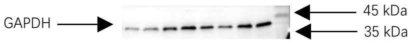

C57BL/6J WB original image

MMP9

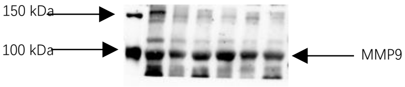

CD44

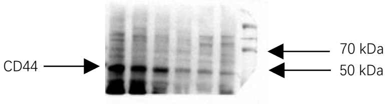

GAPDH

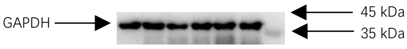

HAS2

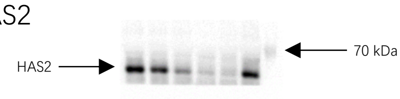

HYAL2

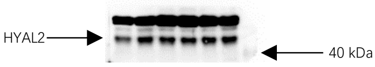

GAPDH

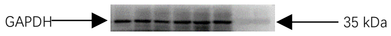

Supplement: Supplementary file 1 [file molecules-28-07449-s001.zip › WB original image.pdf]
