# Supplementary figures and images for: Effects of Radiation-Induced Skin Injury on Hyaluronan Degradation and Its Underlying Mechanisms
Source: Molecules. 2023 Nov 6;28(21):7449. doi: 10.3390/molecules28217449 (PMC10647323; doi:10.3390/molecules28217449)

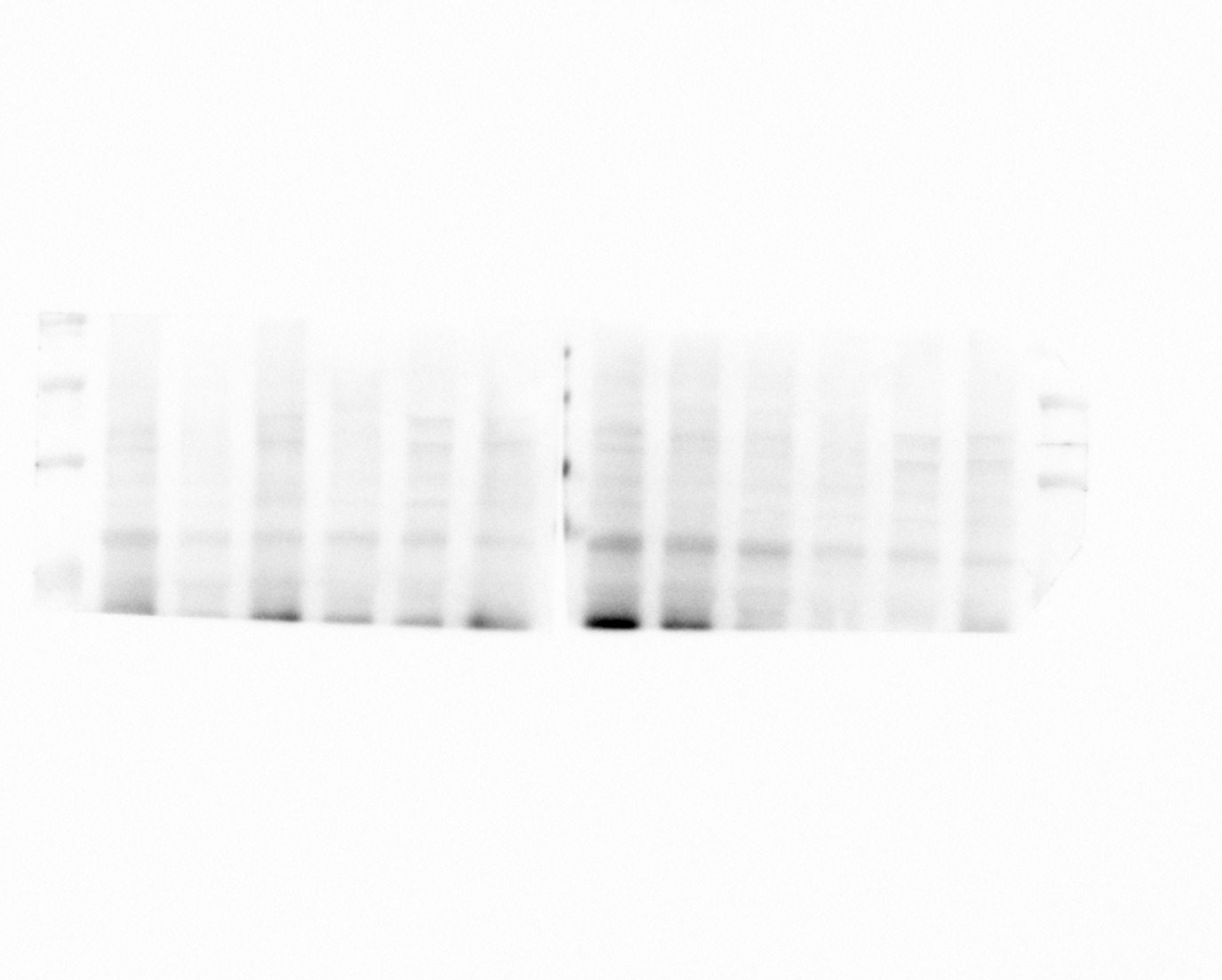

Supplement: Supplementary file 1 [file molecules-28-07449-s001.zip › C57BL6J CD44.Tif]

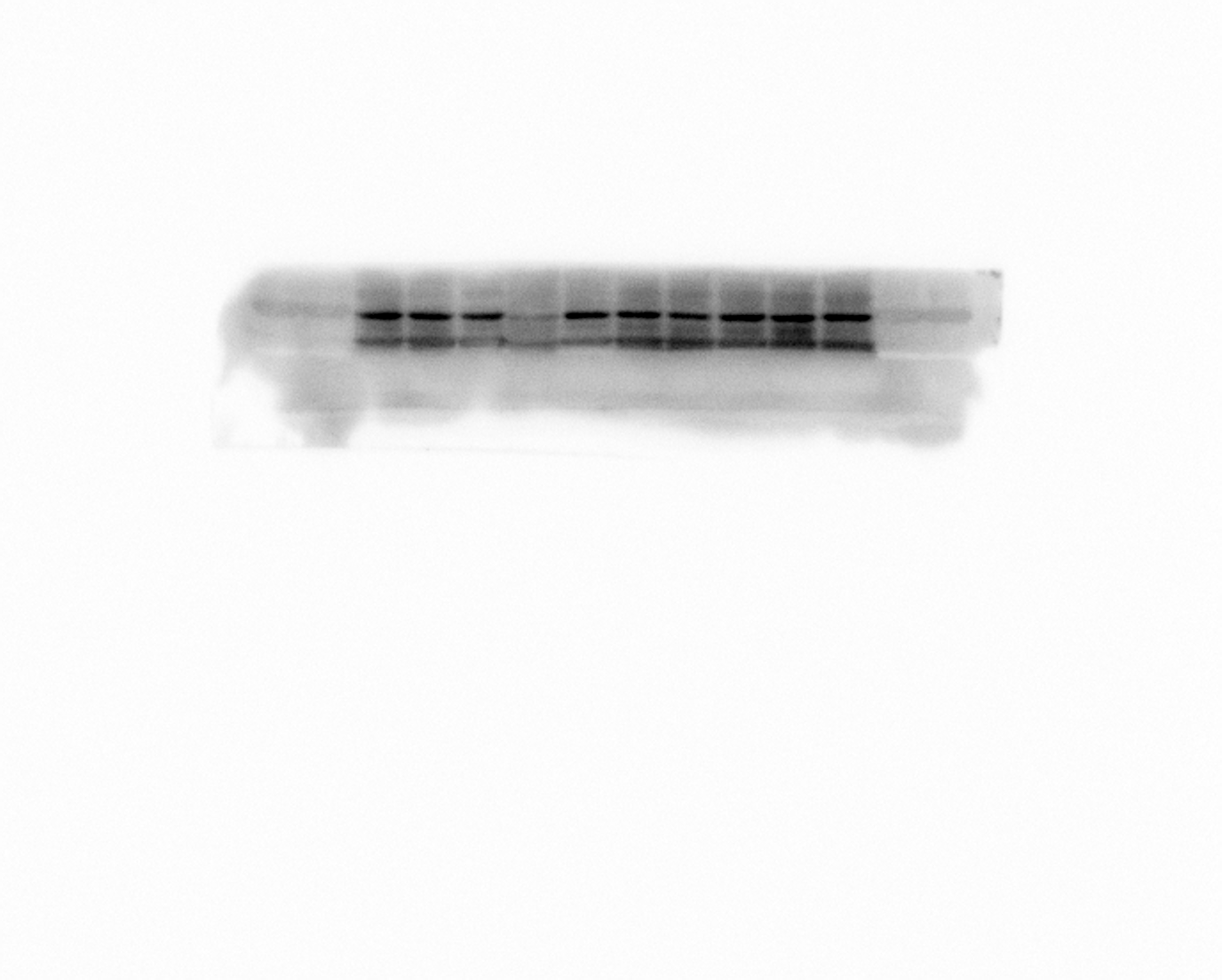

Supplement: Supplementary file 1 [file molecules-28-07449-s001.zip › C57BL6J GAPDH 4.Tif]

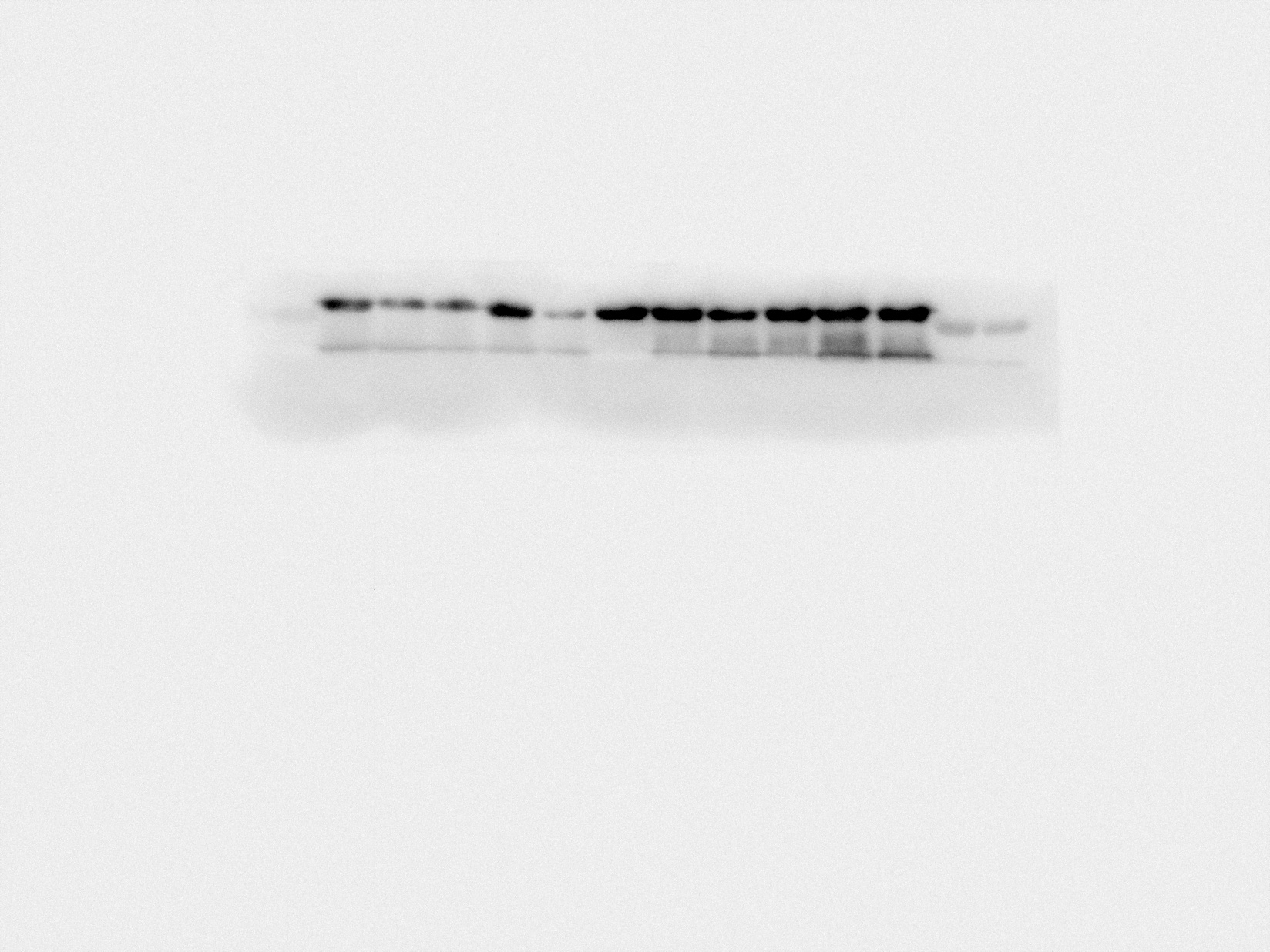

Supplement: Supplementary file 1 [file molecules-28-07449-s001.zip › C57BL6J GAPDH4.jpg]

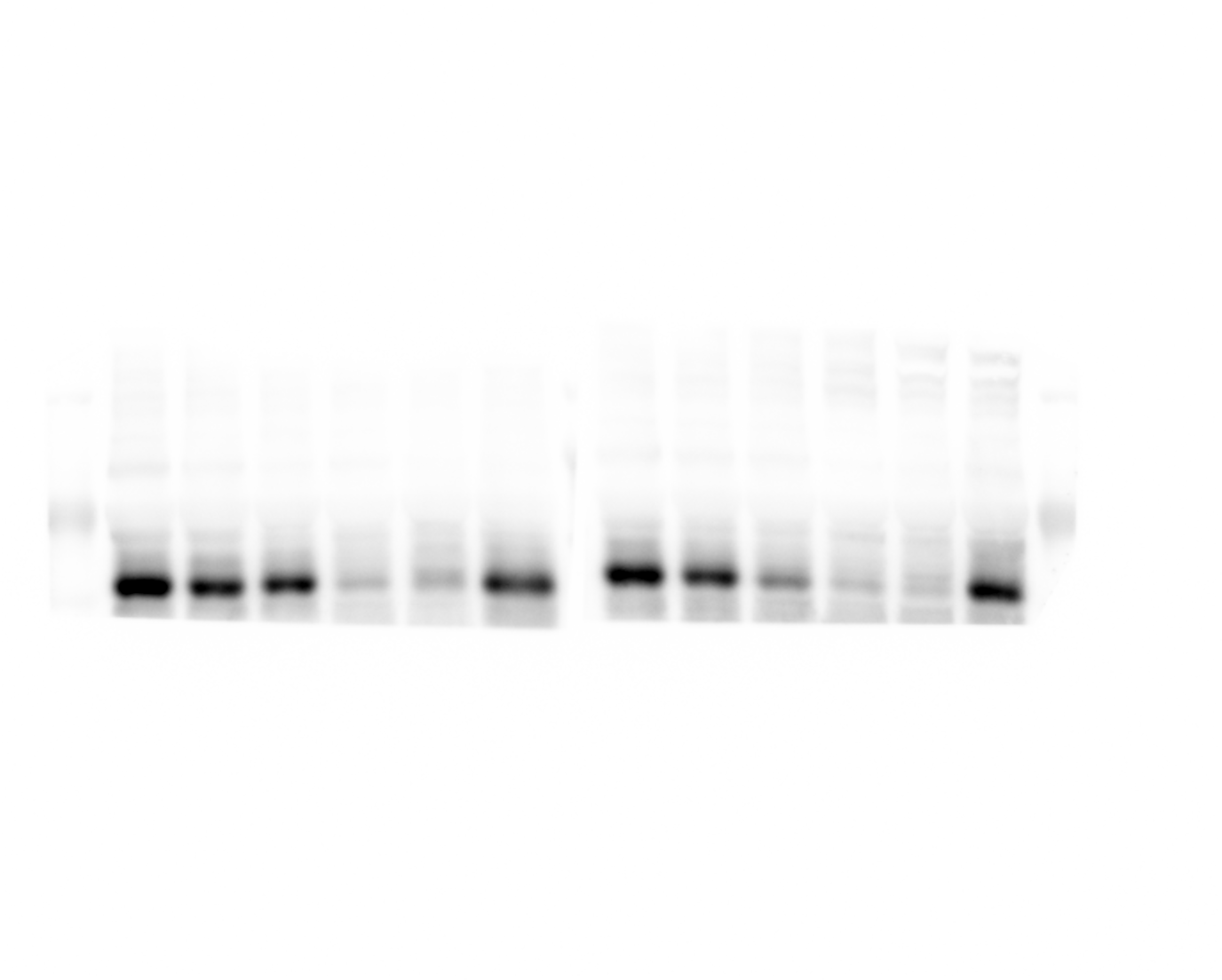

Supplement: Supplementary file 1 [file molecules-28-07449-s001.zip › C57BL6J HAS2.Tif]

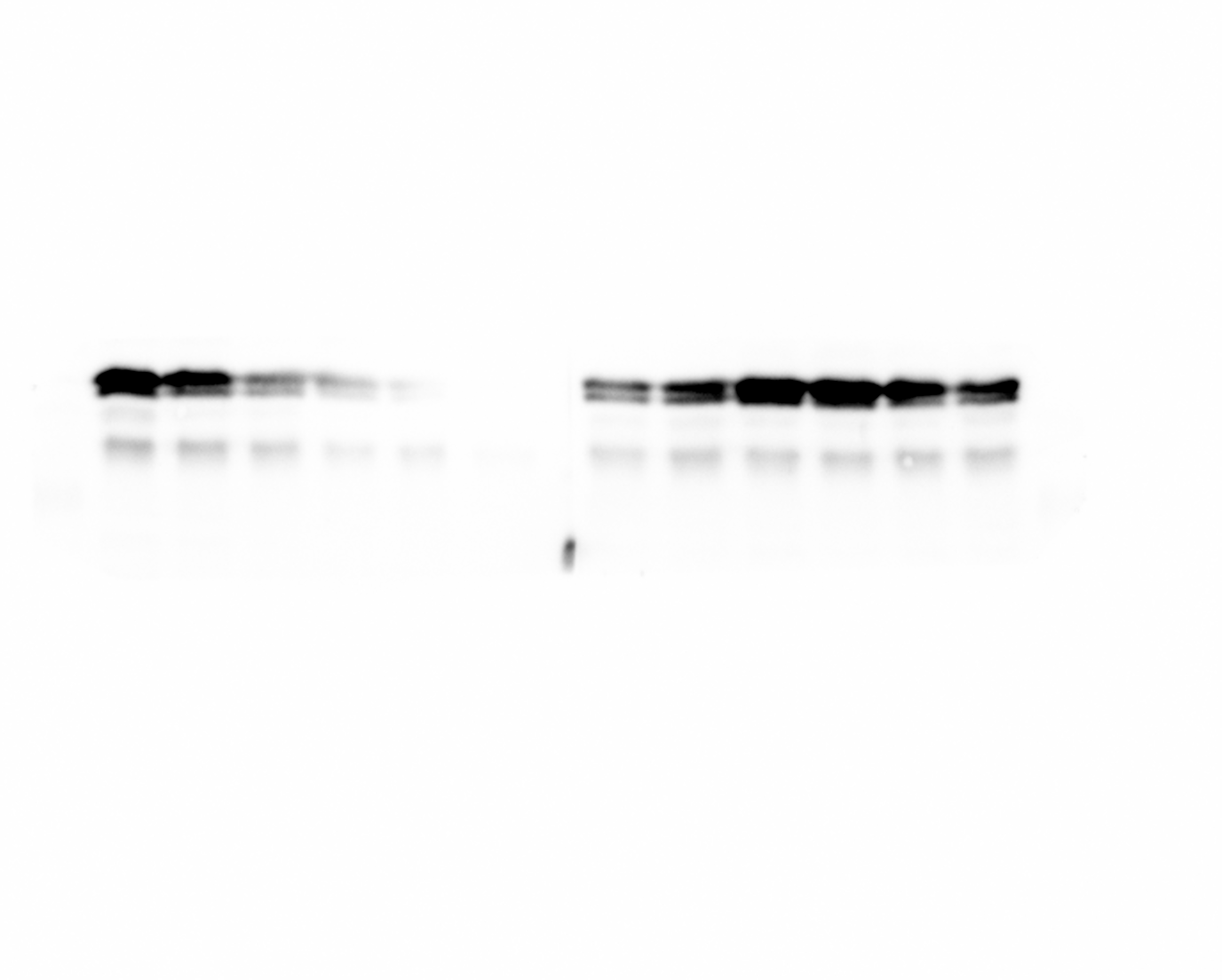

Supplement: Supplementary file 1 [file molecules-28-07449-s001.zip › C57BL6J HYAL2.Tif]

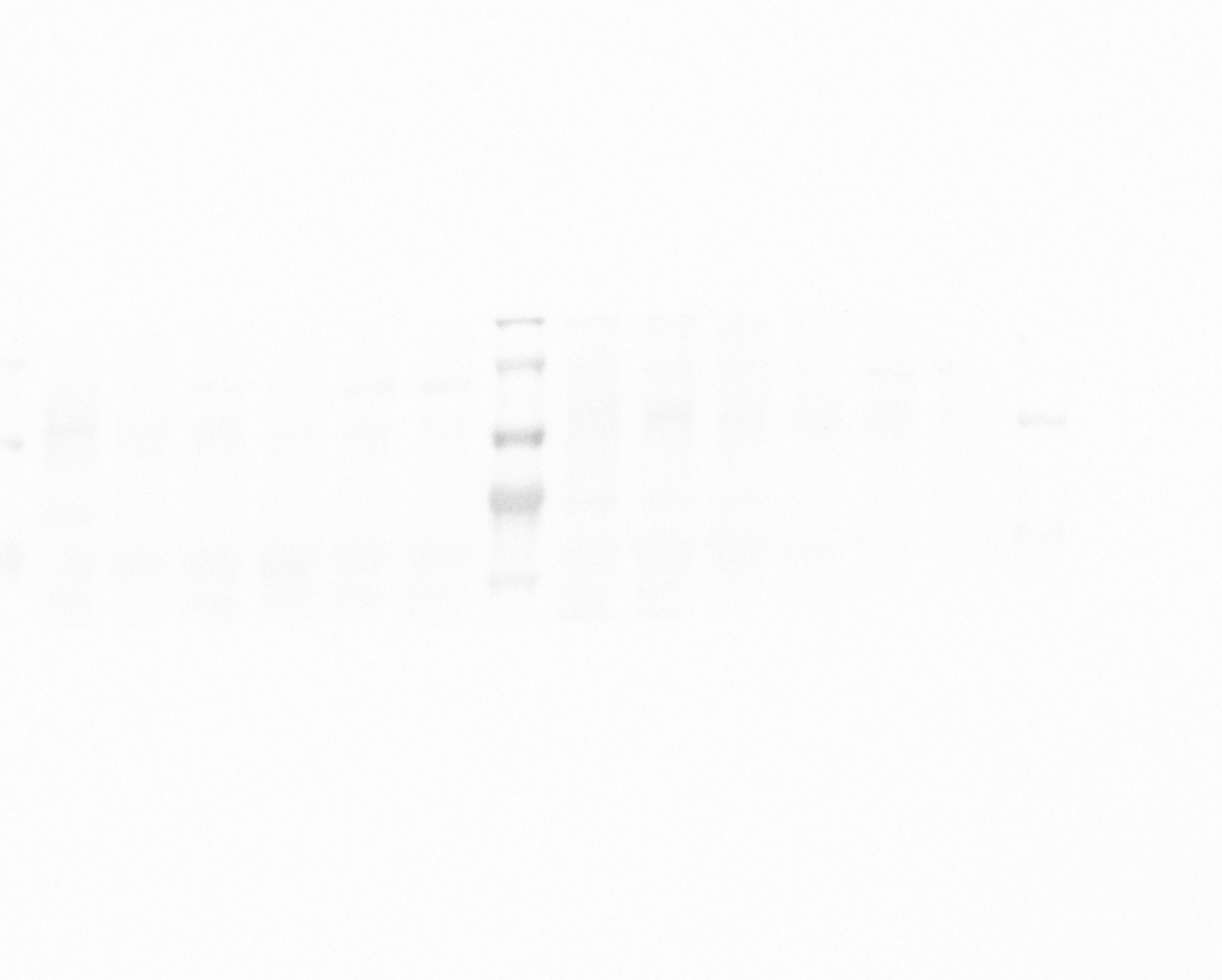

Supplement: Supplementary file 1 [file molecules-28-07449-s001.zip › C57BL6J mmp9.Tif]

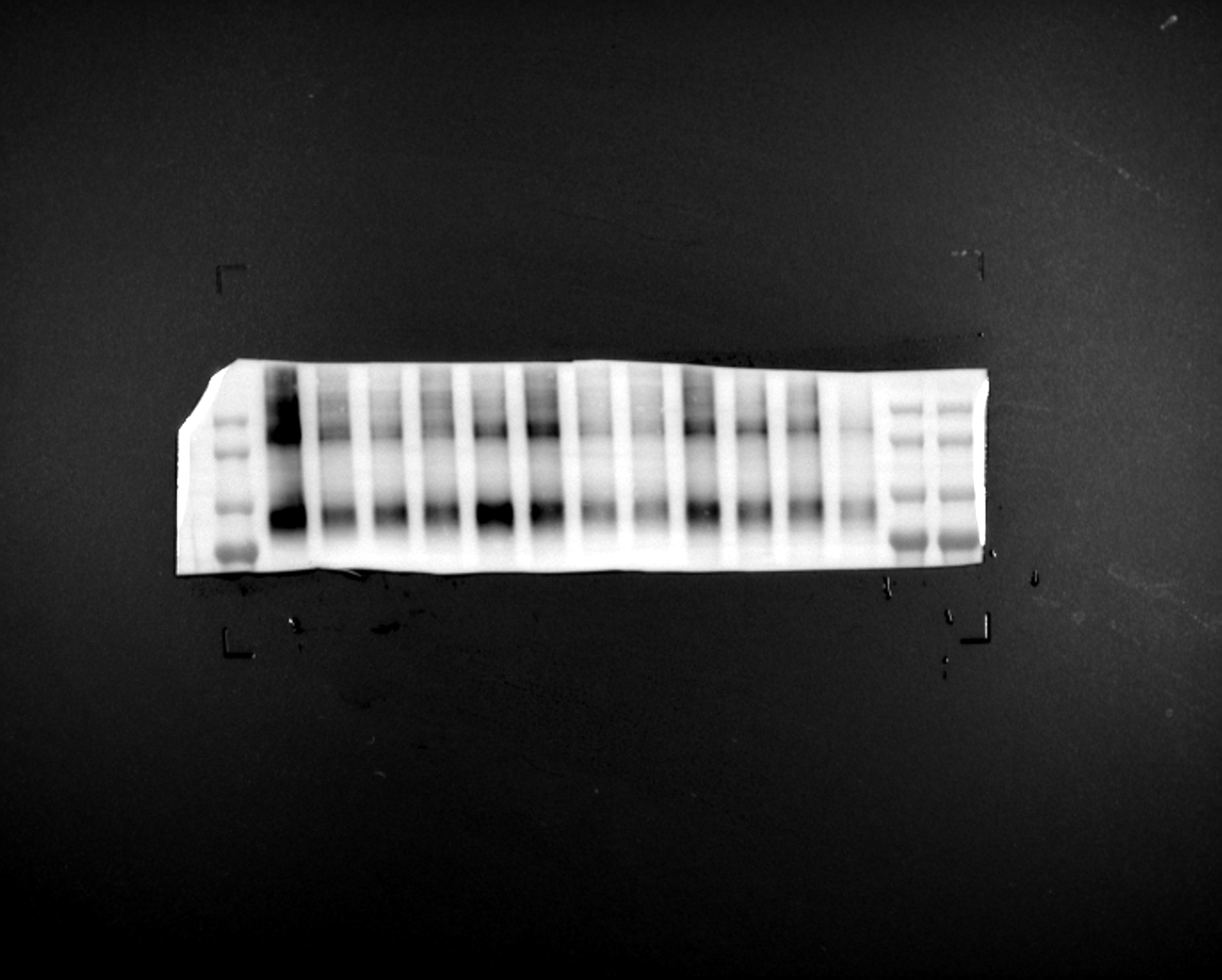

Supplement: Supplementary file 1 [file molecules-28-07449-s001.zip › CD44-membrane 3-original.Tif]

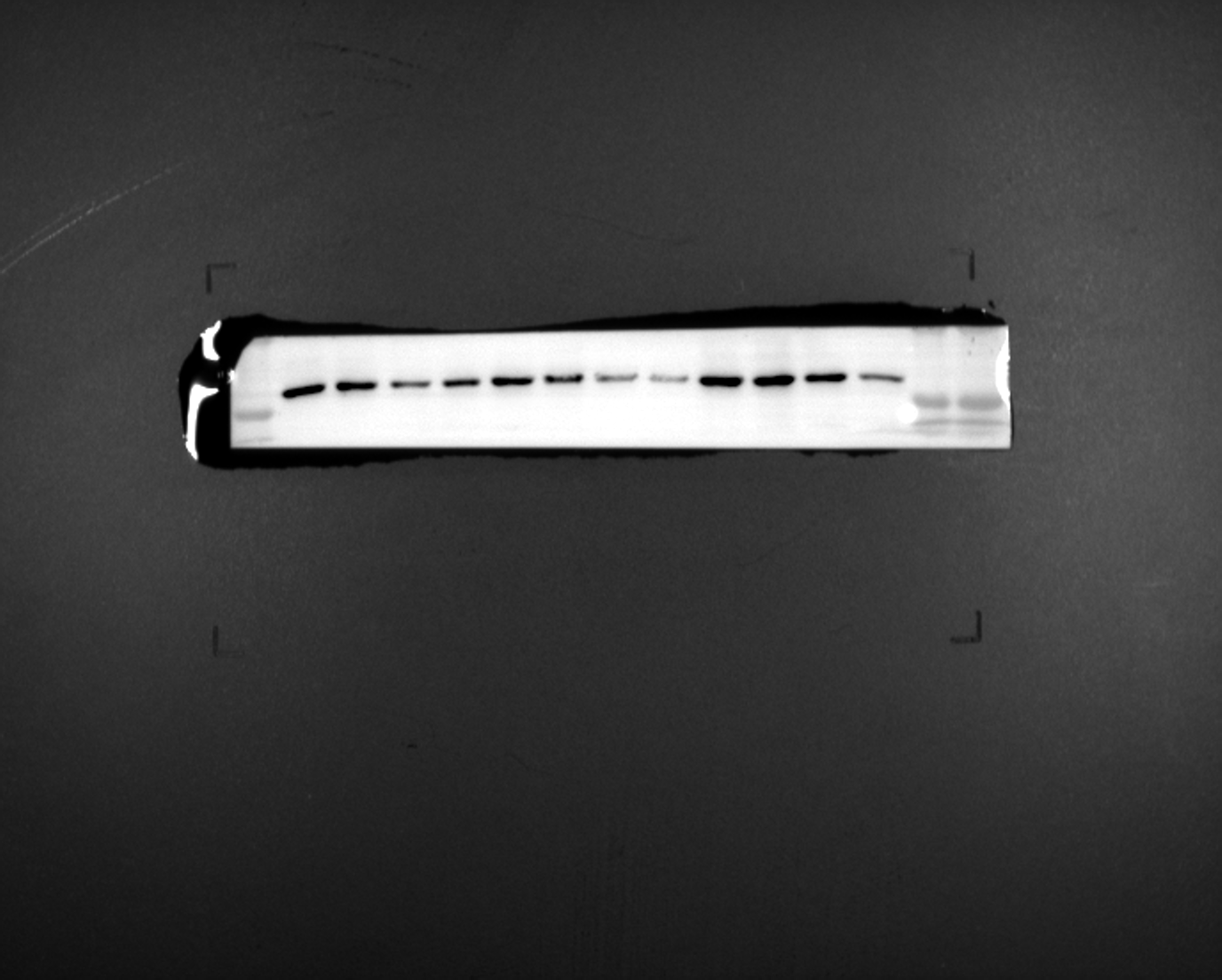

Supplement: Supplementary file 1 [file molecules-28-07449-s001.zip › GAPDH-membrane 3-original.Tif]

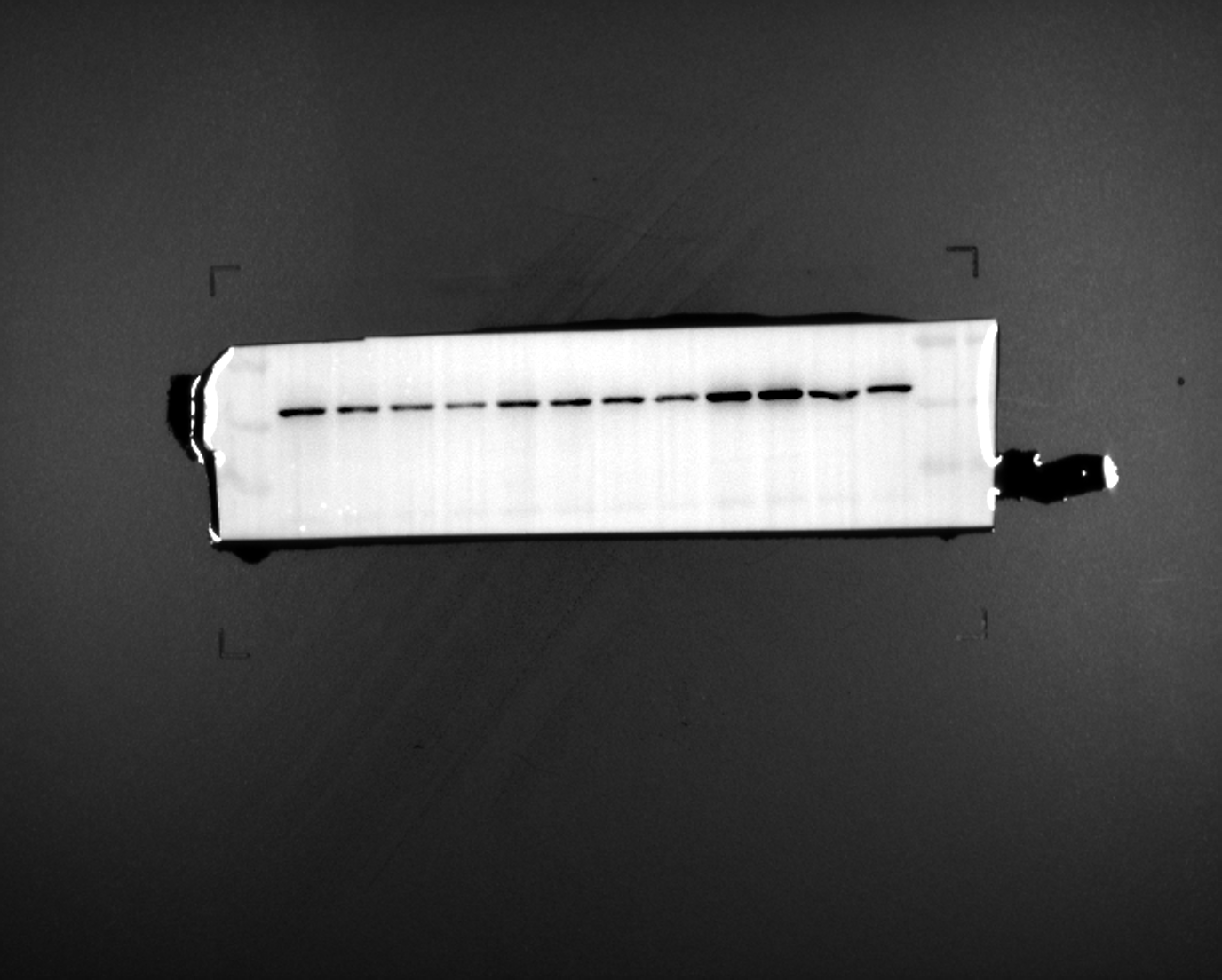

Supplement: Supplementary file 1 [file molecules-28-07449-s001.zip › GAPDH-membrane 4-original.Tif]

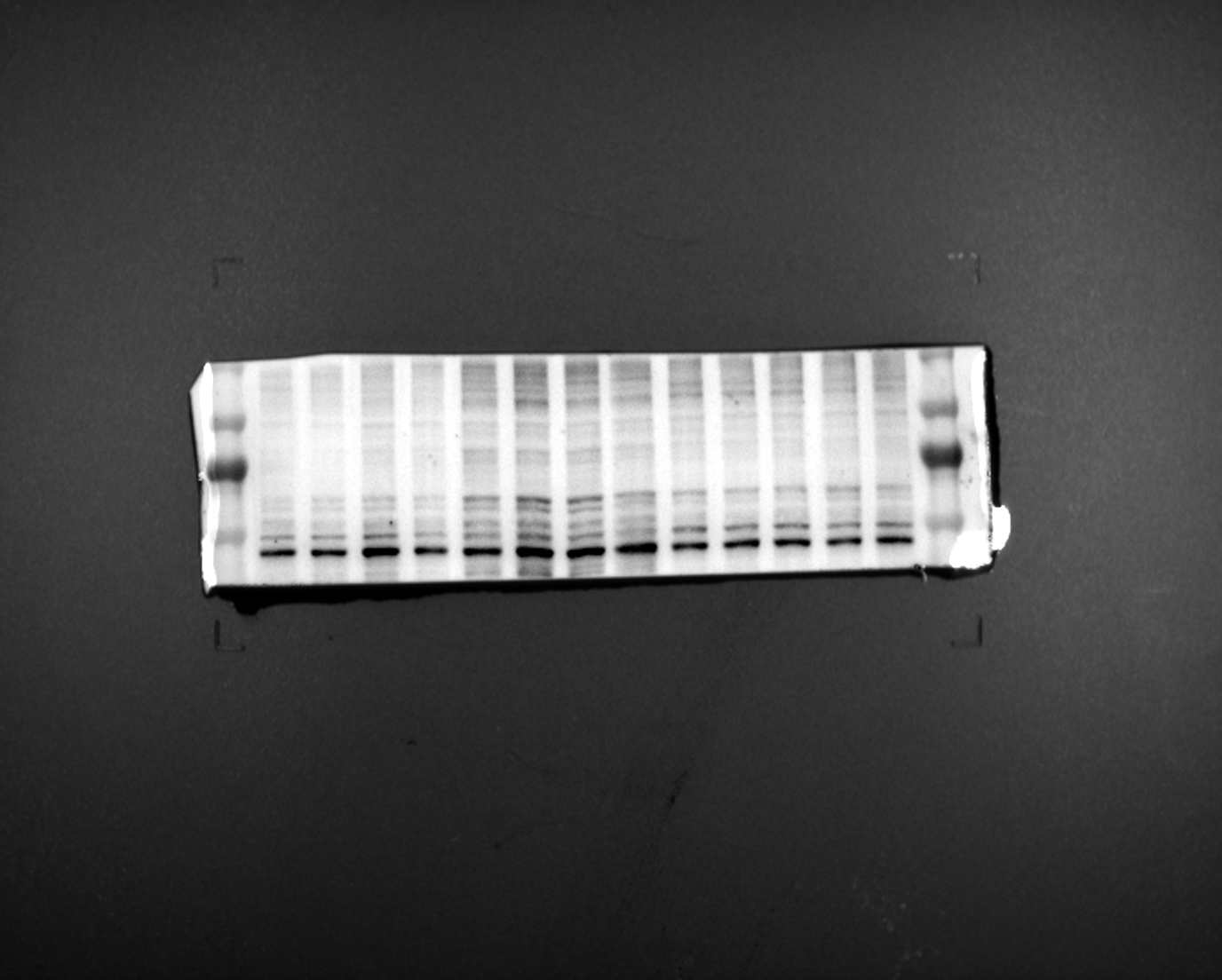

Supplement: Supplementary file 1 [file molecules-28-07449-s001.zip › HAS2-membrane 2-12íó24íó48h-original.Tif]

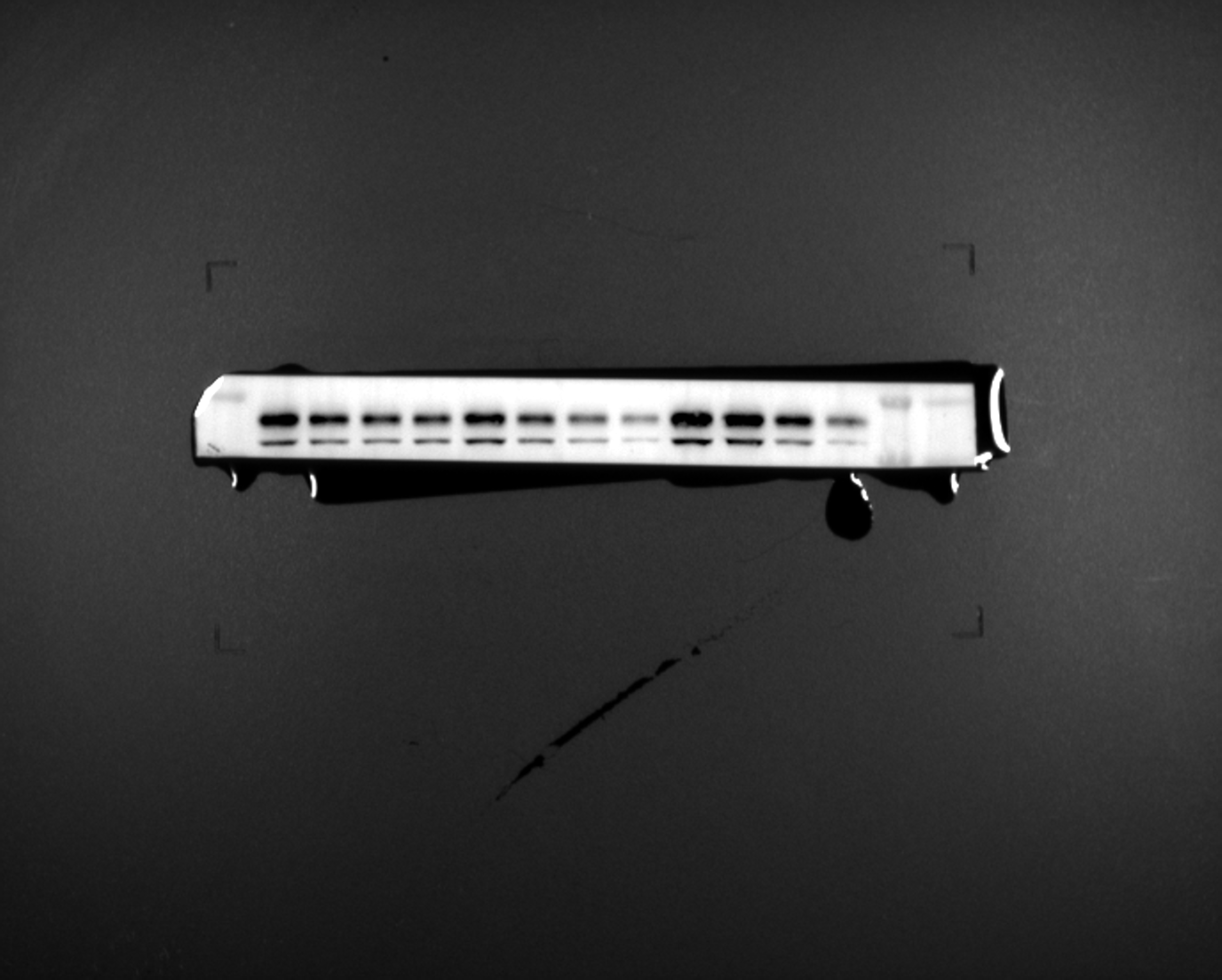

Supplement: Supplementary file 1 [file molecules-28-07449-s001.zip › HYAL2-membrane 4-original.Tif]

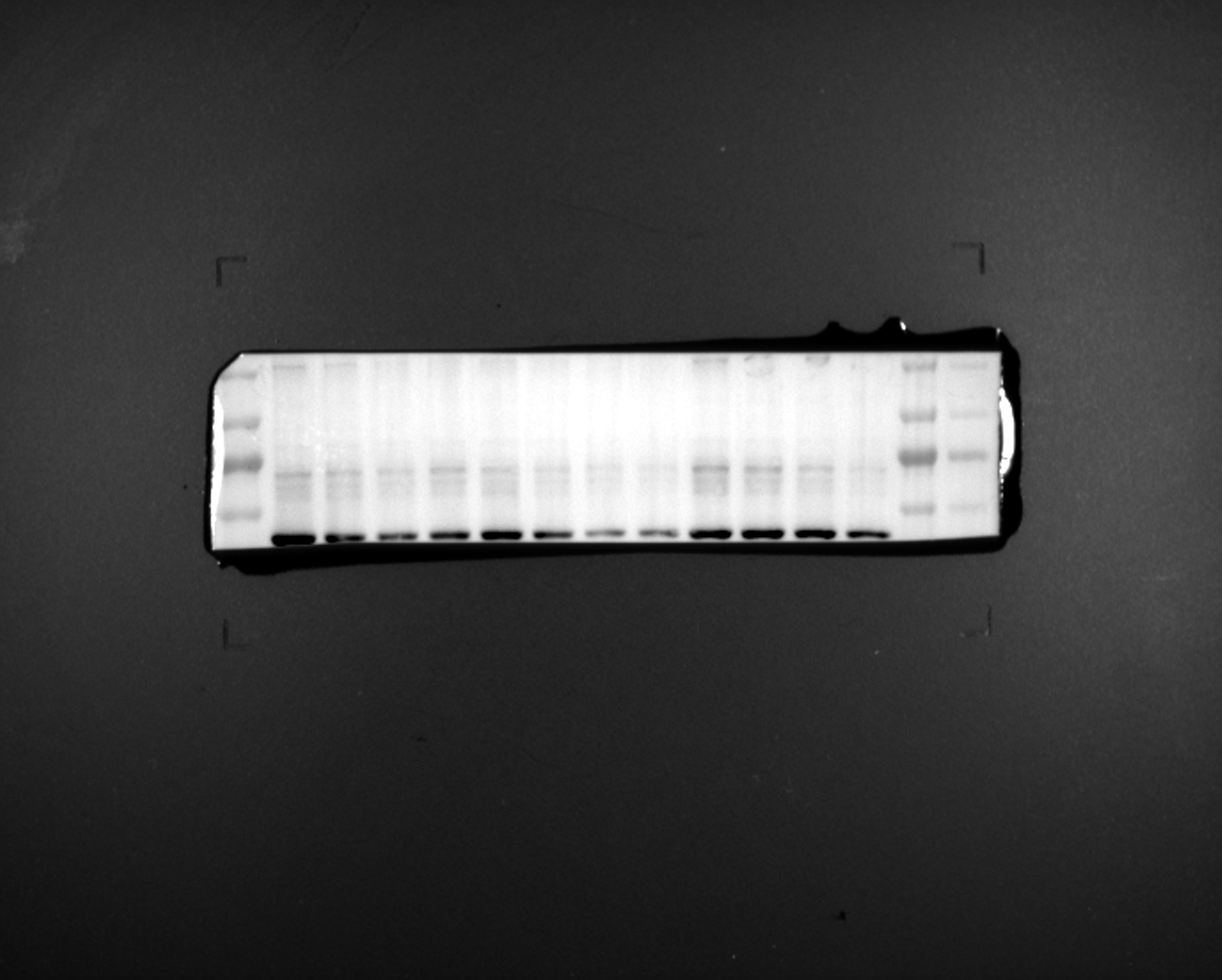

Supplement: Supplementary file 1 [file molecules-28-07449-s001.zip › MMP2-membrane 3-original.Tif]

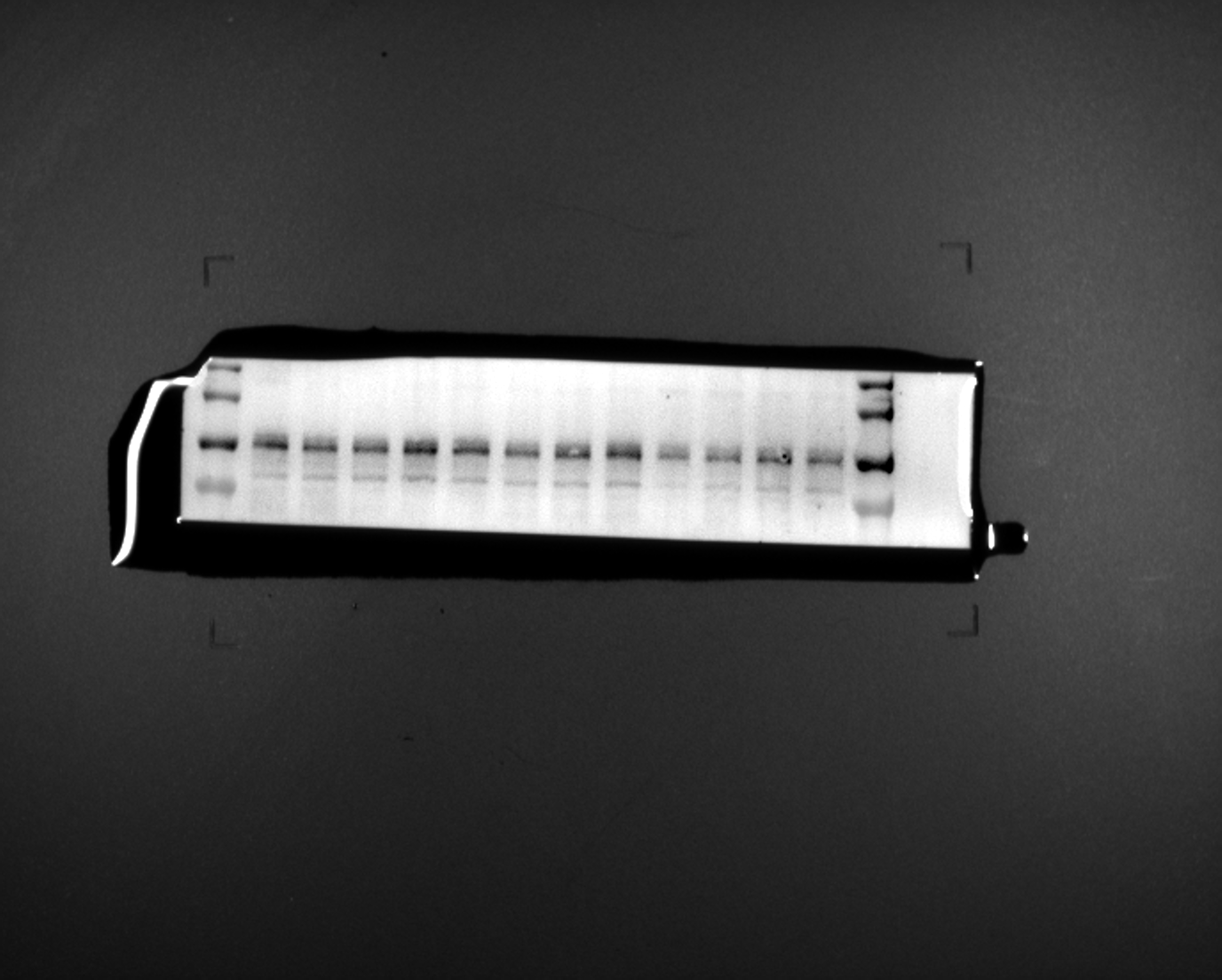

Supplement: Supplementary file 1 [file molecules-28-07449-s001.zip › MMP9-membrane 2-original.Tif]
